# A review on reactive transport model and porosity evolution in the porous media

**DOI:** 10.1007/s11356-022-20466-w

**Published:** 2022-05-06

**Authors:** Yousef Baqer, Xiaohui Chen

**Affiliations:** School of Civil Engineering, University of Leeds, Leeds, LS2 9JT UK

**Keywords:** Reactive, Evolution, Transport, CSH, Nuclear, Geological, Multiphysics

## Abstract

This work comprehensively reviews the equations governing multicomponent flow and reactive transport in porous media on the pore-scale, mesoscale and continuum scale. For each of these approaches, the different numerical schemes for solving the coupled advection–diffusion-reactions equations are presented. The parameters influenced by coupled biological and chemical reactions in evolving porous media are emphasised and defined from a pore-scale perspective. Recent pore-scale studies, which have enhanced the basic understanding of processes that affect and control porous media parameters, are discussed. Subsequently, a summary of the common methods used to describe the transport process, fluid flow, reactive surface area and reaction parameters such as porosity, permeability and tortuosity are reviewed.

## Introduction

Reactive transport in porous media appears in many areas in nature and industrial applications, including subsurface nuclear waste repository, acid fracturing of oil and gas reservoirs, geological carbon storage, geothermal energy systems, seawater intrusion, among others. To model a reactive transport process, it is worth noting that coupled biogeochemist-mechanical multiphysics including advection, diffusion, dispersion and deformation can define the process. This has been mentioned by Tenthorey and Gerald (2006); these reactions affect the properties of the host porous media. In the same vein, Le Gallo et al. (1998), Jin et al. (2013) and Kaszuba et al. (2005) referenced that, with significant alteration of the reaction processes, the feedback mechanisms that influence flow or diffusion through the media can be triggered. More so, Hao et al. (2012a) and Harrison et al. (2017) observed that the reaction process could induce changes in the solid grains and thus affect the reaction rate; dissolution of minerals in an evolving porous media is a typical example. Also, with a substantial dissolution of the minerals, the porosity of the medium will increase. With such an increase in porosity in the porous medium, there are usually secondary effects, especially alteration of its connectivity (Navarre‐Sitchler et al., 2009). The combined alterations can subsequently affect the transport processes by changing the primary transport properties, including permeability, tortuosity, transport mode and pathways.

In the same vein, the system’s reactivity can also be impacted because the dissolution of the mineral can reshape the surface of the dissolving phases or cause the smaller particles in the porous media to completely dissolved, as observed by Noiriel et al. (2009). The dissolution of these minerals is one of the examples of an evolving porous media. During and after its evolution, processes such as precipitation (Gaucher and Blanc, 2006; Chagneau et al., 2015; Brovelli et al., 2009), pore-size alteration (Emmanuel et al., 2010, 2015), bio clogging (Kim and Fogler, 2000; Thullner et al., 2002; Ezeuko et al., 2011), clay swelling (Wang et al., 2014; Herbert et al., 2008) and variations of surface loading and temperature can cause the expansion or contraction of the media (MacQuarrie and Mayer, 2005; Tian et al., 2014, Pfingsten, 2002) and affect the transport properties of porous media. As observed by Houben (2003), biogeochemical reactions account for over 90% of ageing wells. In similar studies, Dauzeres et al. (2010) and Dauzères et al. (2019) observed that the formation of the impermeable calcite layer or any other secondary minerals could be triggered through geochemical interactions at the interfaces of the cementitious materials and clay, and the resulting layer can impede the transport of solute across these interfaces. Understanding these processes is very important, especially for long-term storage of nuclear wastes and mine waste deposits (Soler and Mäder, 2005; Atkinson et al., 1987; Yang et al., 2008; Spycher et al., 2003).

As indicated by Gouze and Coudrain-Ribstein (2002), Jin et al. (2013) and Opolot and Finke (2015), during the evolution of porous media, the transport and chemical properties vary in space and time. Typical examples of this process include soil formation due to weathering and karst formation. In the works of Birk et al. (2003) and Birk et al. (2005), the rates of flow and weathering were identified to affect the evolution of heterogeneity patterns strongly. The interactions between reactive process and flow engineer, the development of the karst, and the dissolution process are further enhanced as the flow rate increases. With increased dissolution, the medium’s porosity and permeability will increase, increasing flow rates, and the cycle continues (Hartmann et al., 2014; Xiao and Jones, 2007). In addition, reactions due to precipitation in natural systems have significant effects on the evolution of these media. With the cementation process reducing the porosity and permeability of the medium while strengthening its mechanical properties, the precipitation reactions can impact the reactivity of the primary minerals and trigger skarn or calcrete formation (Meinert et al., 2003; Wang et al., 1994).

Despite the significant changes to the porous media properties during reactive transport processes, many authors have assumed them to be time-invariant when modelling. But when considering the impact of changes in water content, this simplifying assumption is exempted (Millington and Quirk, 1961). This simplifying assumption that there is no variation in the reactivity and transport parameters in model development is partly because the description of the temporal development of these parameters as a result of changes in the structure and composition of the porous media is quite difficult. Traditionally, reactive transport processes have been modelled using upscaled parameters. With the continuum scale approach, the interdependencies between transport parameters, porosity, reactivity and evolving surface area have been established through some mathematical models (Akanni et al., 1987; Hommel et al., 2018). Both empirical (Low, 1981, Tomadakis and Sotirchos, 1993) and theoretical (Petersen, 1958) formulations have been developed for these relationships.

Studies on evolving porosity and permeability due to reaction in porous media date back to the 1990s, with Steefel and Lasaga (1992), who presented the first numerical paper on the reactive infiltration instability. In a subsequent article, Steefel and Lasaga (1994) found that quartz precipitation impacts the diffusing flow paths over an increasingly wider volume, unlike the reactive worm holing effect caused by the matrix dissolution. In another study by Steefel and Lichtner (1994), they illustrated the impacts of the reactions in the rock matrix on the fracture volume from the surrounding matrix, leading to armouring of the fracture or isolation. Steefel and Lichtner (1998) showed that the balance between the rock cementation and the fracture is delicately balanced, and subsequent evolution of the fracture-rock system hang on it. Navarre-Sitchler et al. (2011) considered the evolution of the pore connectivity and tortuosity on diffusivity. The evolution of the process reactivity has not been addressed in prior reactive modelling efforts. Still, the case involving the dissolution approaches zero reactive surface area has been addressed in the past. However, in a study by Noiriel et al. (2012) on reactive flow with calcite, they concluded that considering the formation of high surface area precipitation when capturing the evolution of the system’s reactivity is very important.

The geochemical reactions and transport processes that control how the porous media evolve occur at the microscale. Moreover, knowing how complex and heterogeneous the pore structure and the multiphysics nature of the problem is, acquiring a representative progression of the effective properties of the media in a macroscale-based approach is challenging. However, with the recently developed imaging techniques such as X-ray microtomography and improved pore-scale model formulations and higher computing power, the exploration of these processes at the pore-scale is now more possible than before from both experimental and numerical simulation. With these developments, recent studies on reactive transport modelling have focused on describing the processes at the microscale. However, one major downside of the pore-scale approach is the difficulty in simulating reactive transport on larger scales. To handle this limitation, hybrid multiscale approaches have been developed.

This article presents a comprehensive review of past and current efforts to understand the underlying physics of reactive flow in porous media. With a brief look into the different modelling scales—pore-scale, mesoscale, continuum and hybrid multiscale approaches are addressed. More so, an extensive study was conducted on the other solution strategies for solving coupled multiphysics problems associated with reactive transport and porosity evolution in porous media.

## Reactive transport in porous media

Reactive transport modelling provides both space and time descriptions of the evolution of the species undergoing both transport process and chemical reactions. The flow process through a porous medium can be described through different modelling scales. The flow through the medium occurs through the void space, which is bounded by the solid skeleton. Changes in the structure of the pore surface or its morphological makeup can happen because of several coupled and interacting processes. While changes in the pore surface properties and its pore’s geometry occur on the microscale or molecular scale, the scale of engineering application is typically larger. Hence, the impacts of the morphological modification of the flow field are often described by the effective hydraulic properties to overcome the computational demand. Based on the continuum modelling approach, the medium’s permeability is often used to measure resistance to flow, while the geometry of the pore is left unresolved. Using permeability as a parameter intrinsically prevents the necessity of describing the interface of the solid and fluid at the cost of losing the microscopic description. To integrate the changes in the structure and morphology of the pore, the permeability is updated using permeability–porosity relations.

### Modelling scales

**Fig. 1:**
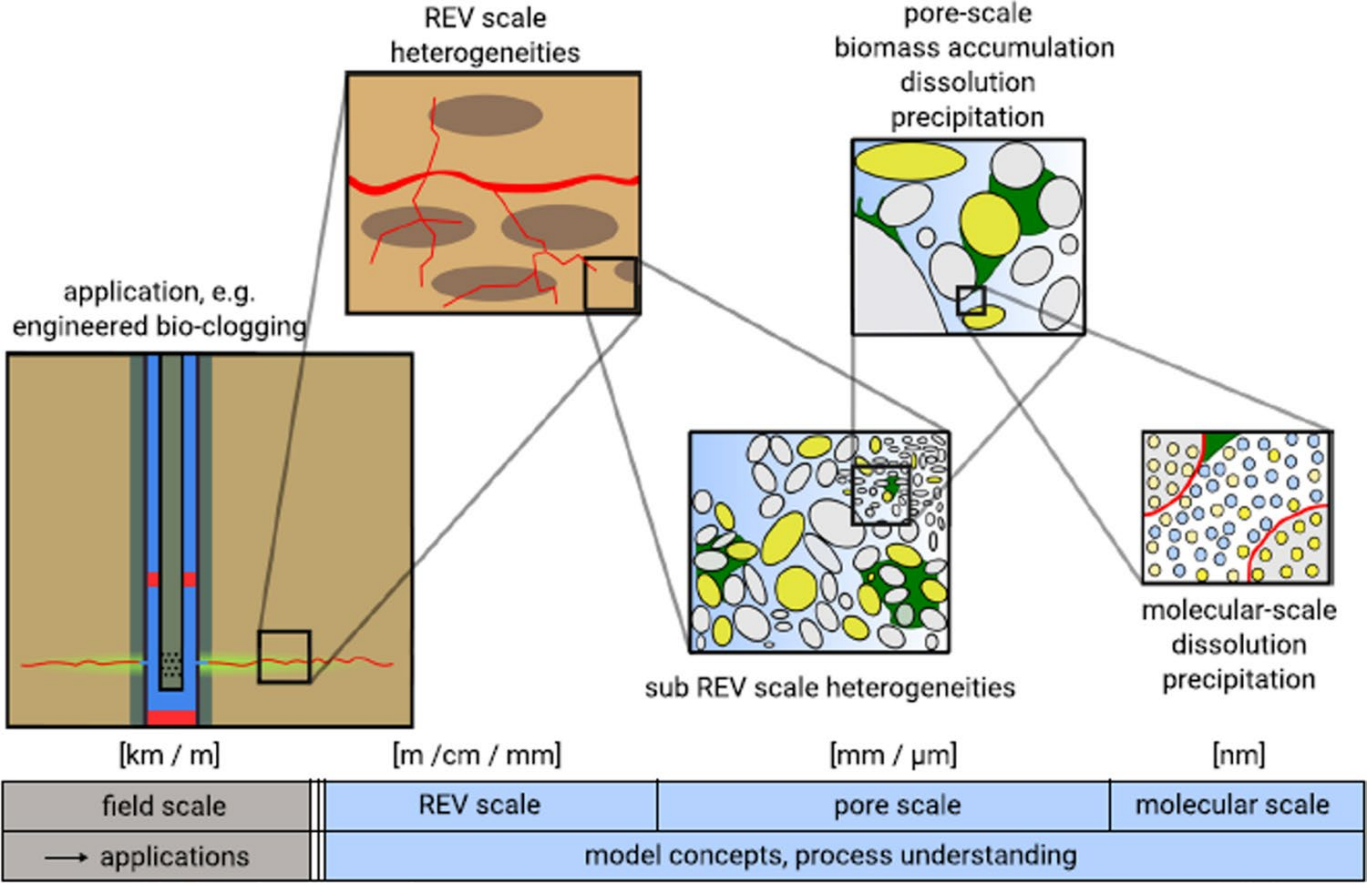
Modelling scales for reactive transport in porous media (Hommel et al., 2018). REV (representative elementary volume)

The hydraulic properties of porous media are linked to the interface between the solid skeleton and the pore space. Although the behaviour of the matrix-pore system is strongly dependent on the interfaces at the pore-scale, the computational cost at this level is too high when simulating domains with large sizes. Using the representative volume element approach helps to characterise spatial scale dependencies. Depending on the property of interest, the required dimensions of the representative volume element may vary (between millimetre and meter range) throughout the porous medium. In Fig. 1, a recursive look at a porous medium with bio clogging is shown. In this figure, each cut-out is a representation of a possible different modelling scale. The choice of the spatial dimension for each scale is arbitrary, and the transiting from one scale to another is done gradually. One benefit of this figure is to show that the details decrease with increasing scale. Although with increasing domain simulation, the computing power required to simulate reactive transport flow is large, and most scientists prefer to adopt the continuum approach, many scientists today are beginning to use the pore-scale approach with the help of supercomputers. Some of these applications are presented in subsequent sections of this paper.

The pore-scale simulation is based on a strong physical foundation. Still, the knowledge of pore geometry is required, which can be challenging to determine, and impractical at scales of higher magnitude than the pore-scale. Although the upscale approaches overcome this limitation, they fail to capture several transport features observed during experiments, including breakthrough curves with long asymmetrical tails (Neuman and Tartakovsky, 2009) and the extent of reactions in mixing-controlled chemical transformations (Knutson et al., 2007; Li et al., 2006). Also, the instability inflows with variable density and the disparity in fractal dimensions of diffusion and dispersion fronts (Måløy et al., 1988). These limitations occur as a result of violations of the closure assumptions on which the continuum approaches are based. Regardless of the upscaling approach such as volume averaging, pore-network models, method of moments and inadequacy in the macroscopic descriptions of pore-scale processes may occur because of homogenisation through multiple-scale expansions and its variations and thermodynamically constrained averaging (Battiato et al., 2009), the nonlinearities of pore-scale governing equations with the boundary conditions require linearisation and other approximations.

Majority of works on upscaling approaches address deriving models for effective properties that show the relationship of microscopic characteristics of the porous medium with macroscopic properties. Such techniques show any existing connections between the physicochemical processes on different scales, provided the governing assumptions are still valid. However, they cannot be applied in identifying the validity of the governing assumptions and the regions of the computation domain where the continuum model fails. This is essential for hybrid models that apply microscale and macroscale descriptions of the same physics in different regions of a computation domain (Tartakovsky et al., 2008b). On the other hand, continuum approaches that depend on characteristic dimensionless numbers can provide quantitative measures to validate various upscaling approximations. For example, the Peclet number determines how well an advection–dispersion equation adequately represents the pore-scale dispersion; the Damkohler number is useful in predicting the breakdown of macroscopic-based models with concurrent pore-scale diffusion and nonlinear homogeneous and linear heterogeneous reactions (Battiato et al., 2009). Moreover, both Damkholer and Peclet numbers can determine if the advection, diffusion and linear heterogeneous reactions in a capillary tube are homogenised.

#### Pore-scale approach

Understanding the processes occurring at the pore-scale is essential for developing predictive models that couple flow, transport and the evolution of the reaction parameters and helps to understand the relationships among these processes and parameters and their influences on the reactive transport at the pore-scale and continuum scale.

Pore-scale simulations have been useful in enhancing the understanding of large-scale natural and man-induced processes. Their significance is due to their capabilities in providing predictions for local transport that is computationally inexpensive and accurate. Simultaneously, they allow for variations of the system’s parameters, such as the geometry of the pore space, fluid properties and boundary conditions, for assessing their impact, which is difficult to achieve in experiments (Meakin and Tartakovsky, 2009). Furthermore, using pore-scale simulations improved continuum transport property assessments by varying the parameters of the pore space structure, thus offering an understanding of the scale of dependence of transport parameters, which macroscale approaches cannot capture. The most common pore-scale modelling methods include lattice Boltzman and smoothed particle hydrodynamics.

With the use of modern and non-invasive imaging techniques, visualising the structure of porous media at pore-scale is now possible. Werth et al. (2010) provided a review of several of these techniques, including nuclear magnetic resonance imaging, X-ray microtomography and optical imaging methods, for hydrogeology and reactive transport applications. 3D X-ray microtomography, especially, is widely used because of its high resolution. The use of these imaging methods allows for visualising pore structures at the submicron resolution, enhancing the view into the complexity of the structure of porous media.

**Fig. 2:**
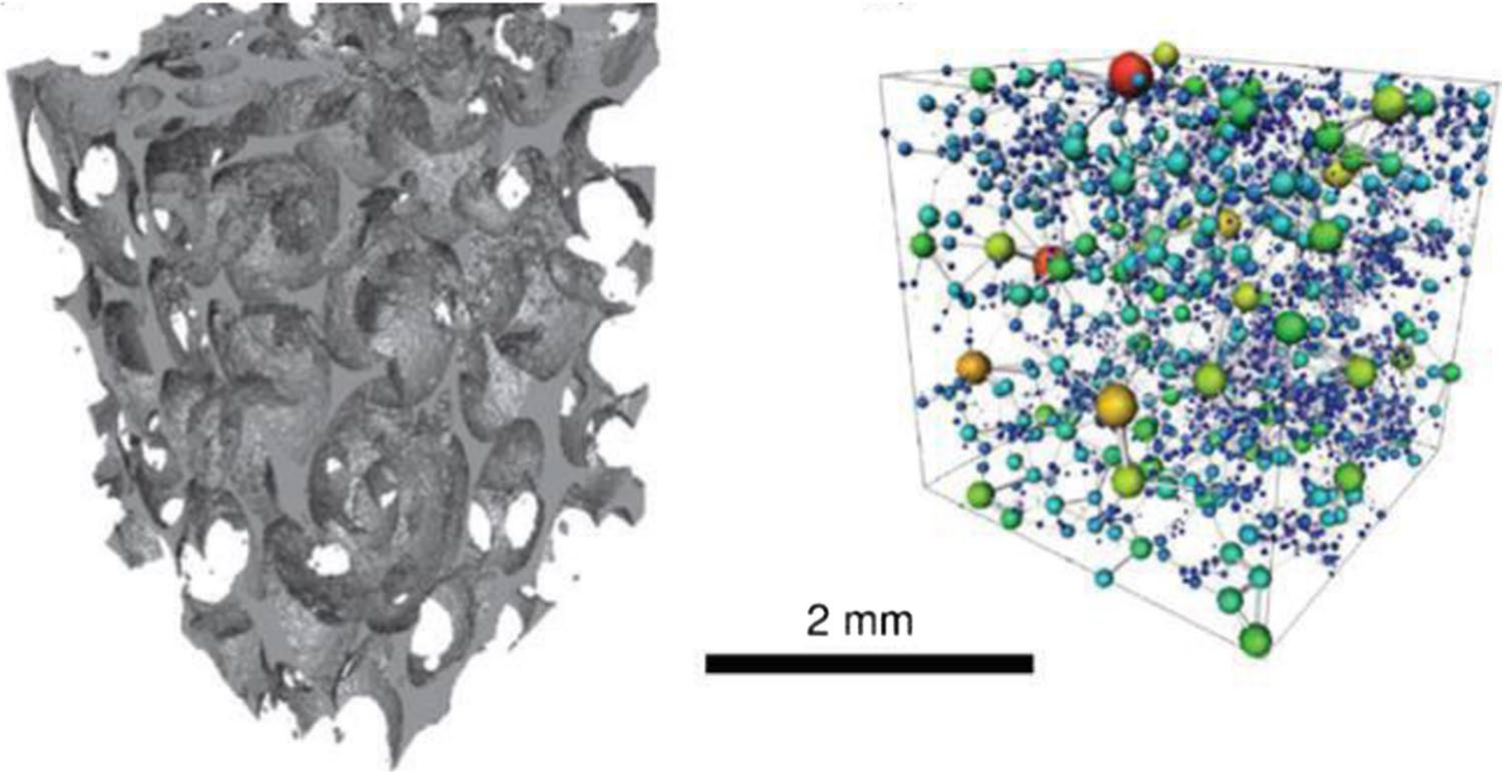
**a** Pore space image of carbonate and **b** the developed pore network model (Blunt et al., 2013)

Figure 2 illustrates a 3D micro-CT (computed tomography) image of a limestone pore space, characterised by large, well-connected pores. These images make constructing the pore network models possible by representing the pores as spheres while the throats of the pores as the connecting cylinders to the pores of the medium (Blunt et al., 2013). However, as mentioned by Navarre‐Sitchler et al. (2009), in some cases, a fraction of the pores are isolated such that connectivity to other surrounding pores is blocked, creating dead ends and no contribution to the effective porosity of the system.

##### Solution strategies for pore-scale approaches

In the pore-scale model, first presented by Bekri et al. (1995), the porous medium is described by void and solid voxels, named the “voxel method”. The local governing equations for the solute concentration are numerically solved through the finite difference method. The evolution of the rock-fluid interfaces is then calculated, and the changes in porosity or permeability are determined. The voxel method is sometimes replaced with a more efficient interface that accurately tracks the algorithms to simulate complex surface motions. The pore-scale model is centred on resolving the Stokes equation and the convection–diffusion equation, accompanied by conditions on the deposition or dissolution flux at the pore walls.

Governing equations are as follows:

For a steady-state flow through a porous medium $\Omega$ with a fluid phase ${\Omega }_{F}$ and a solid phase ${\Omega }_{S}$ separated by an interface $\Gamma$, the Stokes equation in the fluid phase is (Varloteaux et al., 2013):

1 $$\left.\begin{array}{c}\mu {\nabla }^{2}v+\nabla p=0 \, \, \, \, \, \\ \\ \nabla .v=0\end{array}\right\}$$

where $\mu$ is the fluid dynamic viscosity, assumed to be constant, $p$ is the fluid pressure and ${\varvec{v}}$ is the fluid velocity vector. For no-slip conditions at the fluid–solid interface, ${\varvec{v}}=0$. The solute flux is

2 $$J=cv-D\nabla c$$

$D$ is the solute molecular diffusion and $c$ is the volumetric solute concentration.

For no bulk chemical reaction, the local convection–diffusion equation in the fluid phase becomes:

3 $$\frac{\partial c}{\partial t}+\nabla .\left(cv-D\nabla c\right)=0$$

And the boundary condition for the solute concentration at the interface is assumed to be a first-order surface reaction, given as

4 $$n.J=\kappa \left(c-{c}^{*}\right)$$

where $\kappa$ is the local reaction rate constant, $n$ is the unit normal vector and ${c}^{*}$ is the concentration of the solute at equilibrium. The normal displacement to the wall, caused by the reaction, which is proportional to the solute flux at the wall.

5 $$\frac{\partial W}{\partial t}=-{\rm K}_{c}{\rho }_{F}\kappa \left(c-{c}^{*}\right)$$

where ${\rho }_{F}$ is the fluid density and ${K}_{c}$ is the stoichiometric coefficient of the reaction. The velocity of this displacement is taken to be infinitesimal.

A. Level set method (LSM)
  Varloteaux et al. (2013) implemented the level set method (LSM) to simulate the complex surface motions, replacing the voxel method in Bekri et al. (1995). The benefit of the level set method is its ability to deal with curves and surfaces on a fixed Cartesian grid without parametrising the domains. Also, the LSM can track the locus of shapes with changing topology, even when the shapes split into two or more, or developing holes (Sethian and Smereka, 2003). Combining the pore-scale model with LSM yielded accurate result, though demands high computing time and limited pore volumes.

In the LSM, the solid–liquid interface is defined by a length function based on the usual Cartesian grid system. A triangulated surface represents the interface at the zero level of the length/distance function. The interface is defined as the zero level of the contour function $\phi (\mathbf{x},t)$:

6 $$\Gamma =\left\{x|\phi (x,t)=0\right\}$$

Nevertheless, the level set function satisfies the properties $\phi >0$ for phase 1 and $\phi <0$ for phase 2. And the chain derivation rule for $\phi (\mathbf{x},t)$ is:

7 $$\frac{\partial \phi }{\partial t}+\nabla \phi .\frac{d{x}^{^{\prime}}}{dt}=0$$

with unit normal vector pointing outward of the solid phase from the interface. The velocity of the interface is determined according to this relation:

8 $${V}_{i}=\frac{d{x}^{^{\prime}}}{dt}.\frac{\nabla \phi }{\Vert \nabla \phi \Vert }$$

where ${\varvec{x}}$ is the position vector of the interface and $\parallel \parallel$ is a sign showing the magnitude of the corresponding vector. The propagation velocity is related to the dimensionless displacement and Peclet number by

9 $${V}_{i}=\frac{\partial {W}^{^{\prime}}}{\partial {t}^{^{\prime}}}=\text{Pe Da }{c}^{^{\prime}}$$

where ${c}^{^{\prime}}=\frac{c-{c}^{*}}{\langle c\rangle -{c}^{*}}$ is the normalised solute concentration, Pe is the Peclet number and Da is the Damkohler number; noting that Peclet number is a measure of the ratio of advective transport to mass diffusion rate, and Damkohler number is the characteristic residence to the reaction time of a fluid. Hence, the evolution of the level set function is

10 $$\frac{\partial \phi }{\partial t}+{V}_{i}\Vert \nabla \phi \Vert =0$$

for a given initial geometry $\phi (\mathrm{x},t=0)$.

**Fig. 3:**
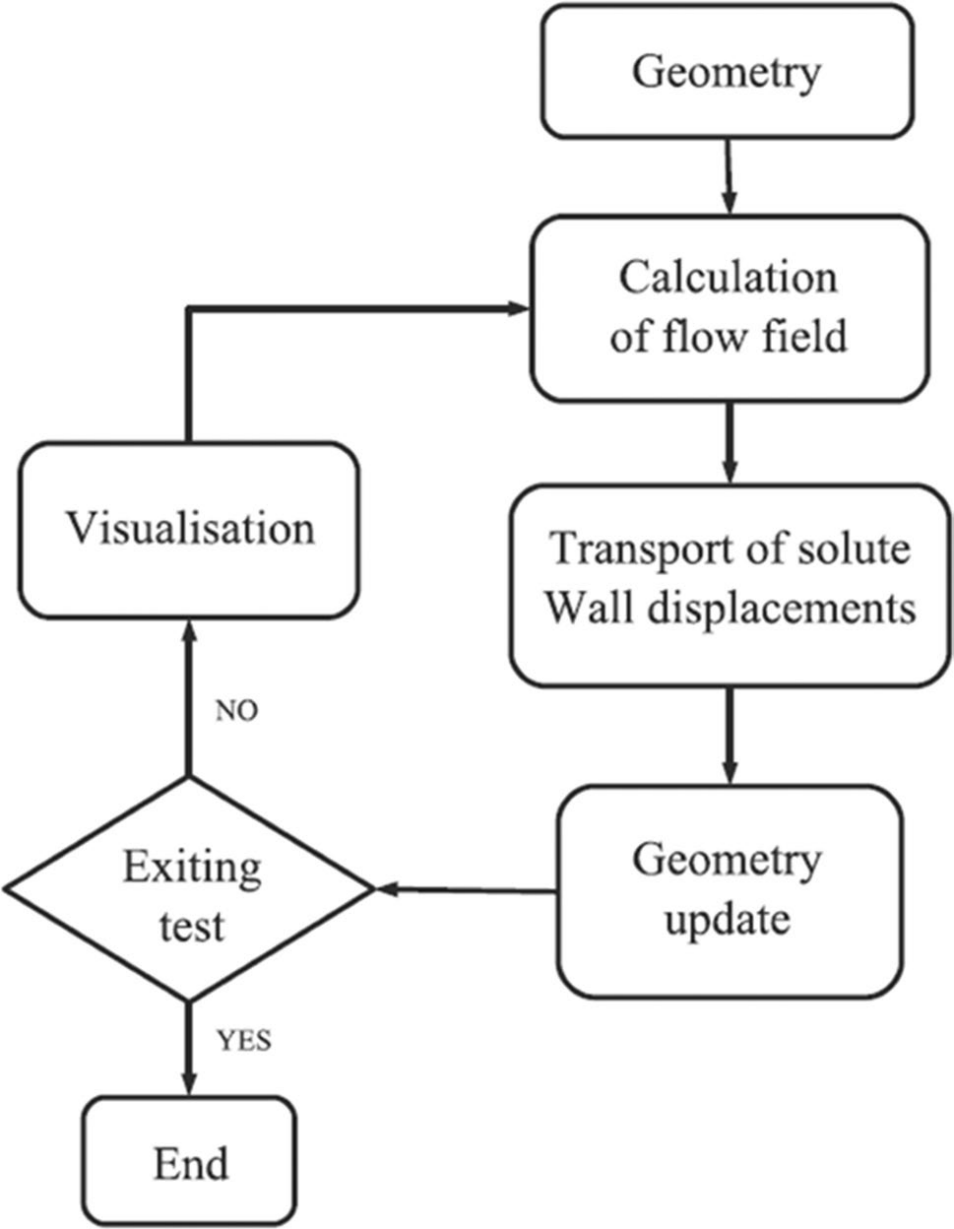
Solution scheme of the reactive transport using pore-scale method combined with LSM (Varloteaux et al., 2013)

Figure 3 shows the solution algorithm implemented by Varloteaux et al. (2013). In the simulation, the coupled Stokes and convection–diffusion equations are solved following the same procedure as Bekri et al. (1995):

- The velocity field ${v}^{n}$ and the concentration field ${c}^{n}$ are estimated with the current interface level set function ${\phi }^{n}$.
- The concentration at the interface is extrapolated from the field ${c}^{n}$ to estimate the interface propagation velocity.
- The new interface ${\phi }^{n+1}$ is then calculated at time ${t}^{n+1}$ using the interface velocity and Eq. (10).
- The new interface is then updated, and the porous medium is visualised.
- The process is repeated until convergence.
- Lattice Boltzmann method
  Many studies have used the lattice Boltzmann method in the simulation of reactive and non-reactive transport in porous media, including dissolution and precipitation processes such as in previous studies (Kang et al., 2006, 2007, 2010; He et al., 2000; Verhaeghe et al., 2006, 2007; Patel et al., 2014). Most of these works, except in Patel et al. (2014), considered heterogeneous reactions at the mineral grain boundary as flux from the boundary, making the separation of reaction and transport computations difficult, and consequently external geochemical codes are coupled. Patel et al. (2014) approach is more efficient because its computation parallelisation is inherently localised and scalable for applications with high computation cost. The heterogeneous reactions were treated as pseudo homogeneous reactions in their work, and they are incorporated as additional source-sink terms in the fluid nodes close to the solid boundary. This technique makes it possible for the sequential operation of transport and reaction step that enables the lattice Boltzmann transport solver to be coupled with the external geochemical algorithm.

Following the model presented by Patel et al. (2014), the fundamental governing equations were extended to multicomponent transport, neglecting the electro-kinetic effects from charged species. The heterogeneous surface reactions that occur at the solid–fluid interface were expressed in terms of the species’ mass balance at the interface:

11 $${J}^{j}. {\left.n\right|}_{\Gamma ={\Gamma }_{s}}={R}_{surf}^{j}$$

where ${R}_{surf}^{j}$ is the surface reaction source-sink term for $j$ component. ${\left.n\right|}_{\Gamma ={\Gamma }_{s}}$ is normal unit vector to the boundary. For all aqueous species, a similar coefficient of diffusion was assumed to limiting the number of transport equations to only primary species. The transport equation for each component was then expressed as the stoichiometric sum of the concentrations of each component’s primary and secondary species.

In the work of Gao et al. (2017b), the mineral reactions were described in a general form, and the rate of kinetic reaction on the reactive surface was proposed as

12 $${R}_{k}^{j}={k}_{r} S \left({\prod }_{j}{C}_{sj}^{nj}\right)\text{ . }\left[1.\frac{Q}{{k}_{eq}}\right]$$

where ${C}_{s}$ is the concentrations of the fluid (gas or liquid) reactants on the mineral reactive surfaces, ${k}_{\mathrm{r}}$ is the reaction rate constant (particular to each reaction), $S$ is the reactive surface of the mineral that is exposed to the fluid reactants, $Q$ is the activity products relating to the concentrations of species and ${k}_{eq}$ is the effective equilibrium constant.

Lasaga (2014) proposed a general form of reaction rates, often used in geochemical simulation, as

13 $${R}_{k}^{j}={k}_{r} S \text{. }{\left[1 {\Omega }_{j}^{\theta }\right]}^{\eta }$$

where the empirical parameters $\theta$ and $\eta$ indicate the dependence of the reaction rate on saturation ratio. For more details on available models for dissolution and reaction rates, the reader is advised to check the works ofMarty et al. (2015), who provided a database of precipitation and dissolution rates of clay minerals, and Bethke (1996), De Simoni et al. (2005), Donado et al. (2009) and Ajayi and Gupta (2019) who provided several models for reaction rate constant, ${k}_{\mathrm{r}}$.

Lattice Boltzmann method solves a discrete set of Boltzmann equations and recovers the advection–diffusion equation, for an incompressible flow, of the form in Eq. (3), but with a sink source. The collision term in the conventional lattice Boltzmann – Bhatnagar Gross Krook (LB-BGK) approach is simplified by a linear term ${\Omega }^{BGK}$ (Bhathnagor et al., 1954), and neglecting the external force, the equation becomes

14 $$\left.\begin{array}{c}{\partial }_{t}{f}_{i}+{e}_{i} \, \text{.} \, \nabla {f}_{i}={\Omega }^{BGK}\left(x,t\right) \, \, \, \\ \\ {\Omega }^{BGK}\left(x,t\right)=-\frac{1}{\tau }\left({f}_{i}-{f}_{i}^{eq}\right)\end{array}\right\}$$

**Fig. 4:**
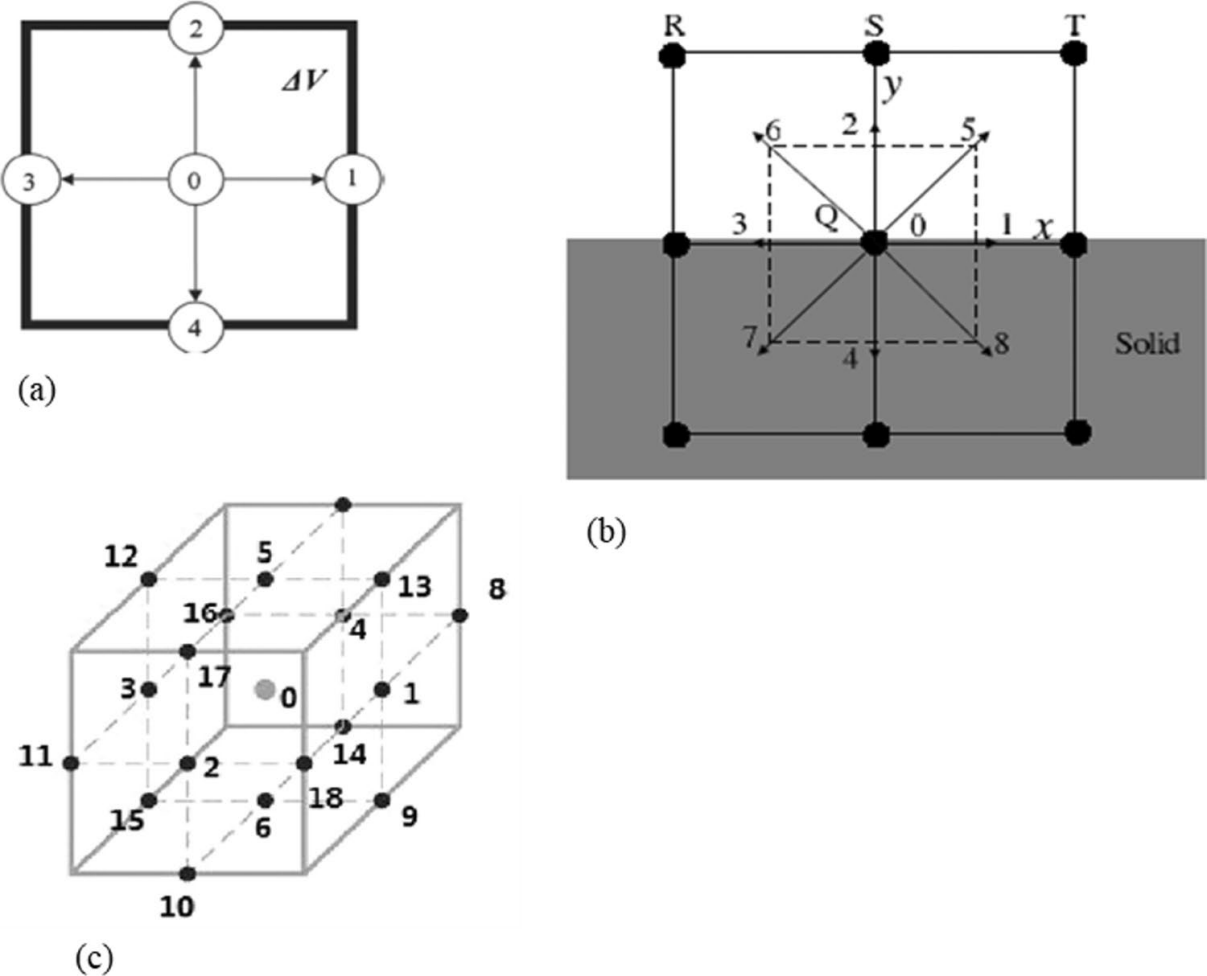
Lattice mesh and nodes **a** for a D2Q5 (Patel et al., 2014), **b** of D2Q4 and D2Q9 (Kang et al., 2007) and **c** for D3Q19 (Gao et al., 2017a)

**Fig. 5:**
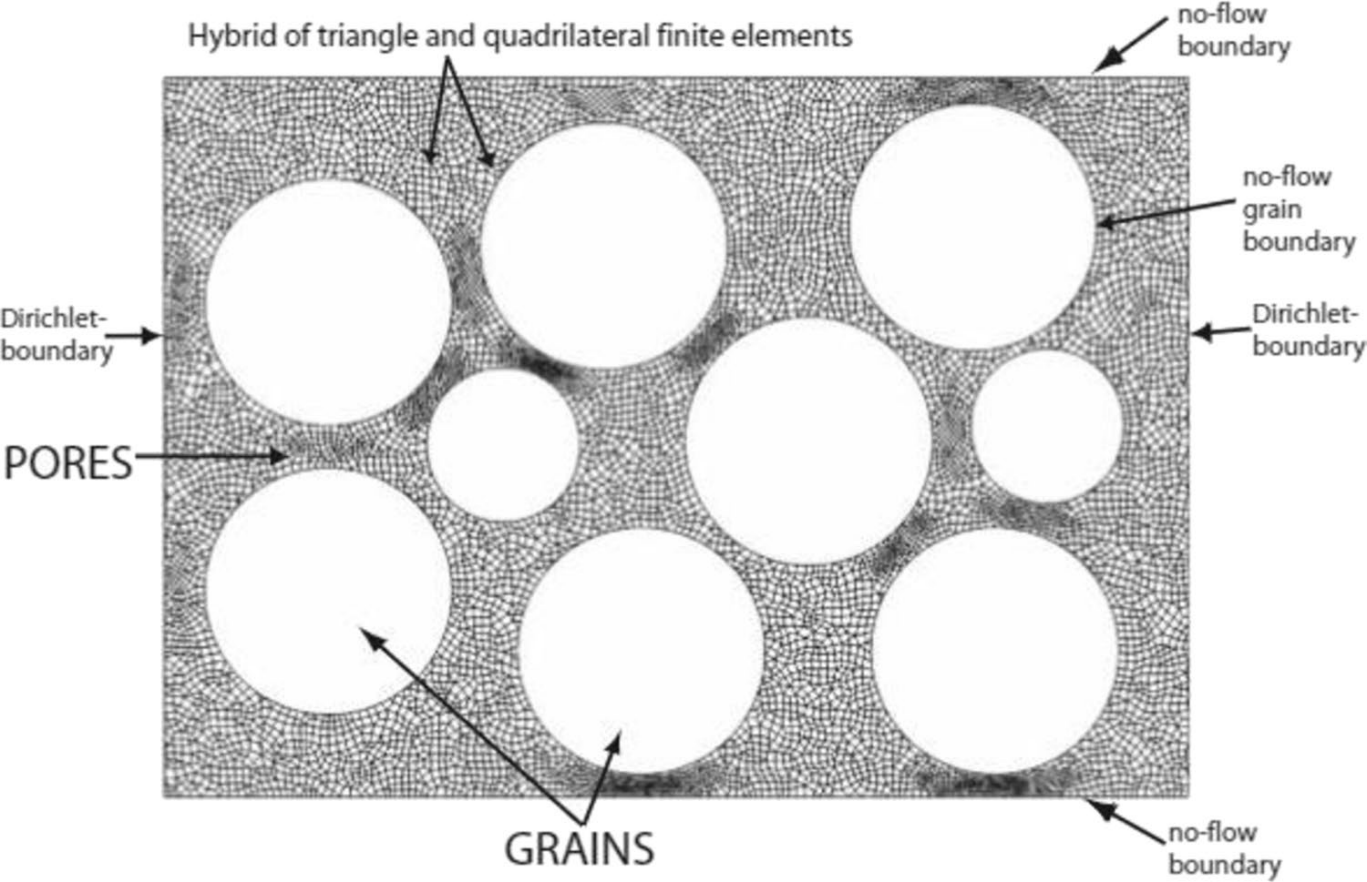
An unstructured FEM with adaptive refinement mesh having triangular and quadrilateral elements for 2D pore-scale modelling of flow in porous media (Akanji and Matthai, 2010)

${f}_{i}$ represents the probability distribution function for a given particle along a velocity direction with unit vector ${e}_{i}$ in the $i$ th direction dependent on the lattice type. $\tau$ is the relaxation time, and ${f}_{i}^{eq}$ is the equilibrium distribution function. He and Luo (1997) further discretised Eq. (14) in space and time, and for passive scalar multicomponent mass transport. The applicable distribution function for each of the primary species is

15 $$\left.\begin{array}{c}{\mathrm{f}}_{\mathrm{i}}^{\mathrm{j}}\left(\mathrm{x}+{\mathrm{e}}_{\mathrm{i}}\mathrm{\Delta t},\mathrm{t}+\mathrm{\Delta t}\right)={\mathrm{f}}_{\mathrm{i}}^{\mathrm{j}}\left(\mathrm{x},\mathrm{t}\right){\Omega }^{\mathrm{BGK}}\left(\mathrm{x},\mathrm{t}\right) \, \, \, \\ \\ {\Omega }^{\mathrm{BGK}}\left(\mathrm{x},\mathrm{t}\right)=-\frac{\mathrm{\Delta t}}{\uptau }\left({\mathrm{f}}_{\mathrm{i}}^{\mathrm{j}}\left(\mathrm{x},\mathrm{t}\right)-{\mathrm{f}}_{\mathrm{i}}^{\mathrm{eq},\mathrm{j}}\left(\mathrm{x},\mathrm{t}\right)\right)\end{array}\right\}$$

where $\Delta t$ is the discrete-time step. The selection of the form of the equilibrium distribution function and the lattice type is dependent on the system of equations being solved. In the advection–diffusion equation being solved by Patel et al. (2014), an orthogonal lattice D2Q5 (2-dimensional domain with 5 separate velocities) was sufficient to satisfy the isotropy requirement for a 2D geometry Fig. 4a. The corresponding discrete velocity vector for this lattice was given as

16 $${e}_{i} =\left\{ \begin{array}{c} o, \, i=0\\ \left(\mathit{cos}\frac{\left(i-1\right)\pi }{2},\mathit{sin}\frac{\left(i-1\right)\pi }{2}\right)c, \, i=\mathrm{1,2},\mathrm{3,4}\end{array}\right.$$

where $e=\Delta x/\Delta t$, and $\Delta x$ is the distance between two lattice nodes. As referenced in Fig. 4b, c and 5 other forms of lattice mesh have been used by other authors. But the D2Q9 lattice is widely used in the numerical solution of the advection–diffusion equation (Hiorth et al., 2013; Kang et al., 2006; He et al., 2000; Verhaeghe et al., 2006, 2007). Moreover, for low grid Peclet number (Pe < 10), a D2Q5 lattice can also be used. Meanwhile, for a higher Peclet number (Pe > 10), the D2Q5 lattice is less accurate than D2Q9; nevertheless, its stability range is higher. Another key advantage of the D2Q5 lattice grid is that it requires less storage memory and computing time than the D2Q9 lattice, which can significantly gain performance for multicomponent transport simulations with large chemical species.

The equilibrium distribution function for first-order approximation to solve advection–diffusion equation is sufficient, given by Flekkøy (1993) and Patel et al. (2014) as

17 $${f}_{i}^{\;eq,j}\left(x,t\right) ={w}_{i}{C}^{j} \left(1+\frac{{e}_{i}\text{ . }u}{{e}_{s}^{2}}\right)$$

where ${w}_{i}$ are the weights for the distribution function along the $i$ th direction and ${e}_{s}$ is the lattice pseudo-speed. The reader can check the works of Patel et al. (2014), d’Humieres and Lallemand (1987), He and Luo (1997), Ziegler (1993), Kang et al. (2006), Kang et al. (2007), Kang et al. (2010), He et al. (2000), Verhaeghe et al. (2006), Verhaeghe et al. (2007) and Hiorth et al. (2013) for more details on this implementation.

III. Finite element methods
  In Discontinuous Galerkin (DG) finite element methods, the approximation of the solutions to differential equations is made through discontinuous piecewise polynomials, such that the boundary conditions are imposed weakly through bilinear forms. As explained by Sun and Wheeler (2006a) and demonstrated by other authors (Oden et al., 1998, Arnold, 1982, Rivière et al., 2001, Cockburn et al., 2000, Schötzau and Schwab, 2000, Schötzau et al., 2003, Chen and Chen, 2004, Larson and Niklasson, 2004, Karakashian and Pascal, 2003), the DG methods have gained popularity, despite having larger degrees of freedom than conforming approaches, because they (1) are conservative element-wise; (2) support unstructured meshes, variable degrees of local approximations and other nonconforming spaces; (3) easily adaptable, allow for sharp posterior error indicators and local errors; (4) have insignificant numerical diffusion; (5) are applicable in addressing problems and discontinuities with rough coefficients in numerical simulations; (6) are robust and do not oscillate when there are high gradients; (7) are able to deliver exponential rates of convergence with the suitable meshing scheme; (8) are excellent in parallelisation because of the localised nature of their data communications; and (9) provide substantial computational advantage when using explicit time integrations because their mass matrices are block diagonal for time-dependent problems. More so, by simply extending the flux average on the element faces, this method will provide a continuous flux field defined throughout the entire computation domain and efficiently allow coupling with conforming methods.

The simulation of reactive transport in porous media using adaptive DG has been observed to effectively track the moving concentration fronts (Sun and Wheeler, 2006a, 2006b, Sun, 2003). Four primal DG methods have been implemented: Oden Babuska Baumann- DG (OBB-DG) approach by Oden et al. (1998), Symmetric Interior Penalty Galerkin formulation (SIPG) by Wheeler (1978), Nonsymmetric Interior Penalty Galerkin method (NIPG) by Rivière et al. (2001) and Riviere (2000) and Incomplete Interior Penalty Galerkin (IIPG) (Sun, 2003, Dawson et al., 2004). The general formulation of the governing equation, with a source term, is presented below for completeness; the reader is advised to see the referenced works for more details about these schemes.

18 $${\left(\frac{\partial \phi c}{\partial t},N\right)}_{E}- {\int }_{E}\left(vc-D\left(v\right)\nabla c\right)\text{ . }\nabla N+{\int }_{\partial E}\left(vc-D\left(v\right)\nabla c\right)\text{ . }{n}_{\partial E}N={\int }_{E}q{c}^{*}N+r\left(c\right) N$$

where $\phi$ is porosity, Darcy velocity,is the unit normal vector on element$N$ is the weighting function,  is the reaction term and $q{c}^{*}$ is the source term.

IV. Smoothed particle hydrodynamics (SPH)
  This is one of the Lagrangian methods, which is a meshless discretisation of partial differential equations. In this approach, the discretisation of the computational domain with a set of points and the corresponding discretisation scheme is used for discretising the scalar or vector fields as functions of their values at these discrete points. The meshless discretisation scheme permits the discretisation points to be moved with the fluid velocity, even for non-uniform flow. A recent study (Tartakovsky et al., 2016) discretised the Navier–Stokes (NS) and advection–diffusion equations for flow and reactive transport problems using the SPH discretisation scheme. In the Lagrangian coordinate system, the solution to the momentum conservation equation is simplified if the nonlinear inertia term is absent. Given a velocity field, discretising the advective term in the advection–diffusion equation will not cause numerical dispersion. Each discretised point with associated mass and density is assumed as the centre of the fluid particle. This discretisation scheme helps to reduce the Navier–Stokes equations to a system of ordinary differential equations for Newtonian particle dynamics. The sum of the forces between a particle and its neighbours is computed to determine the total force acting on each SPH particle. Hence, fluid–solid interactions can be easily achieved by adding a pair of molecular-like interaction forces to the force terms obtained after the SPH discretisation of the NS equations. For readers interested in using the SPH approach for modelling coupled flow and reactive transport problems, the work of Tartakovsky et al. (2016) is worth reading.

The SPH discretisation of the advective-diffusion–reaction equation of $M$ species, subject to an appropriate initial condition and the Robin boundary condition${D}^{l}\mathbf{n} . \nabla {c}^{l}={g}^{l}({c}^{1},\dots , {c}^{M}, {\Xi }^{1},\dots ,{\Xi }^{L})$, presented by the authors are:

19 $$\frac{D{m}_{i}{c}_{i}^{l}}{Dt}=\sum\limits_{k\in {\Omega }_{p}}\frac{{D}^{I}\left({m}_{i}{n}_{i}+{m}_{k}{n}_{k}\right) \left({c}_{i}^{l}-{c}_{k}^{l}\right)}{{n}_{i}{n}_{k} {\left({r}_{i}-{r}_{k}\right)}^{2}}\left({r}_{i}-{r}_{k}\right)\text{ . }\nabla W({r}_{ik},h) -{m}_{i}\sum\limits_{k\in {\Omega }_{s}}{g}^{l}\frac{2\left({n}_{i}+{n}_{k}\right)}{{n}_{i}+{n}_{k}}\text{. }\nabla W({r}_{ik},h$$

where ${\mathrm{r}}^{l}$ and ${\mathrm{g}}^{l}$ are the homogeneous and heterogeneous reaction rates of species $l$, respectively. ${m}_{i}$ and ${n}_{i}$ are mass and particle density of particle $i$, respectively. ${m}_{k}$ and ${n}_{k}$ are mass and particle density of particle $k$, respectively. $W$ is the SPH smoothing kernel, and ${\Xi }^{l}$ is the dimensionless surface concentration of species $l$ formed on the surface.${r}_{i}$ is position vector of particle $i$, and ${r}_{k}$ is the position vector of the particle *k*. *h* is a small increment in distance. $\sum_{k\in {\Omega }_{p}}$ and $\sum_{k\in {\Omega }_{s}}$ are summations over all the fluid and solid particles, respectively. The interface evolves through the precipitation/accumulation of the surface species with the normal velocity. The precipitation and dissolution of the reaction product can be determined by tracking the masses of the solid, changing according to this equation:

20 $$\frac{d{m}_{i}}{dt}=-{m}_{i}\left({C}^{A,0}+{C}^{B,0}\right)\sum\limits_{k\in {\Omega }_{s}}{g}^{l}\frac{2\left({n}_{i}+{n}_{k}\right)}{{n}_{i}+{n}_{k}}\text{. }{\nabla }_{i}W\left({r}_{i}-{r}_{k},h\right)$$

For a reaction $A+B\to {C}_{s}$, resulting in the formation of a solid phase $C$. ${C}^{l}$ is the mass fraction and ${C}^{l,0}$ is the initial mass fraction, for species $l=A,B$. To determine the new solid particle position during precipitation, the closest fluid particle to the solid particle is replaced with a solid particle. And for dissolution, the solid particles with mass less than or equal to zero is replaced with fluid particles. Finally, the velocity of the newly formed fluid particle is determined using the SPH interpolation scheme ${v}_{i}=\sum_{k\in f+s}\frac{{v}_{k}}{{n}_{k}}W({r}_{ik},h)$, and the concentrations are set to ${C}_{k}^{A}={C}_{k}^{B}=\sqrt{{K}_{sp}}$, where ${K}_{sp}$ is the solubility product. Tartakovsky et al. (2007a), Tartakovsky et al. (2007b) and Tartakovsky et al. (2008a) used a similar formulation to model the precipitation of calcium carbonate for the reaction between calcium chloride and sodium carbonate.

In modelling complex transport processes, the use of the SPH method has some advantages. One of them is the triviality of treating interfacial problems compared with grid-based methods. This means that different fluid phases can be represented using different particle types. Also, within this context, the equations for the advection, diffusion and reactions in the Lagrangian framework are reduced to diffusion and reaction equations, as the SPH fluid particles will advect the solute. Consequently, numerical diffusion is eliminated because of the discretisation of the advection term.

One of the limitations of the SPH method is its high computation cost compared to the grid-based methods. In discretising the spatial derivatives, more bordering particles participate in the computation than the grid-based methods. Another limitation is that SPH schemes may not be able to use higher-order discretisation schemes like the grid-based methods. Hence, the grid-based methods have higher accuracy and computational efficiency than SPH schemes for simple linear problems. But for multiphase/multicomponent reactive transport problems, the SPH method may provide a superior advantage over the grid-based methods (finite element and finite volume). The use of adaptive resolution and consistent discretisation of the spatial derivatives can further enhance the accuracy of the SPH method.

#### Mesoscale approaches

The standard macroscopic equations are not yet needed at the mesoscale because the fluid flow and solute transport processes are simulated at the pore scale, and the exact dynamics of the particle dynamics are not considered. To do this, first, a dummy representation of the porous medium is constructed, which consists of the pore bodies and throats of differing but connected geometries. Then, the possibility of simulating fluid flow and/or reactive transport of interest at the mesoscale through the network is expected, with the implementation of the pertinent physics pore-to-pore.

Pore network modelling (PNM).

The first network model was constructed by Fatt (1956), who used the analogue between flow in porous media and a random resistor network. Since his presentation, the modelling approach has been improved upon the work of other researchers, including Balhoff and Wheeler (2009), Lopez et al. (2003), Blunt et al. (2002), Ryazanov et al. (2009) and Yiotis et al. (2006). It captures irregular lattices, arbitrary wettability, wetting layer-flow and reactive transport.

PNM provides the description of the flow and transport on the mesoscale because of the need to upscale from small-imaged size to larger domains. In this approach, the void space is approximated by a bonds network and nodes with an idealised geometry. In this simplified representation, the pore bodies are spherical, while the pore throats are represented as circular, square, or triangular channels. The difference between the nodes and pore throats and their corresponding simplified geometries eases the complexity and allows analytical or semi-analytical solution methods.

The representation of the actual pore space using both topological and geometrical characteristics strongly influences the simulation accuracy for each application. Generally, the different experimental methods for characterising the pore space can limit constructing the representative pore network. However, previous attempts demonstrated the importance of having a good representation of the geometrical properties of the porous media, including the locations of the pores and throats and the size and shape distributions of the pores and throats (Knackstedt et al., 1998; Bryant and Blunt, 1992, Oren, 1994).

**Fig. 6:**
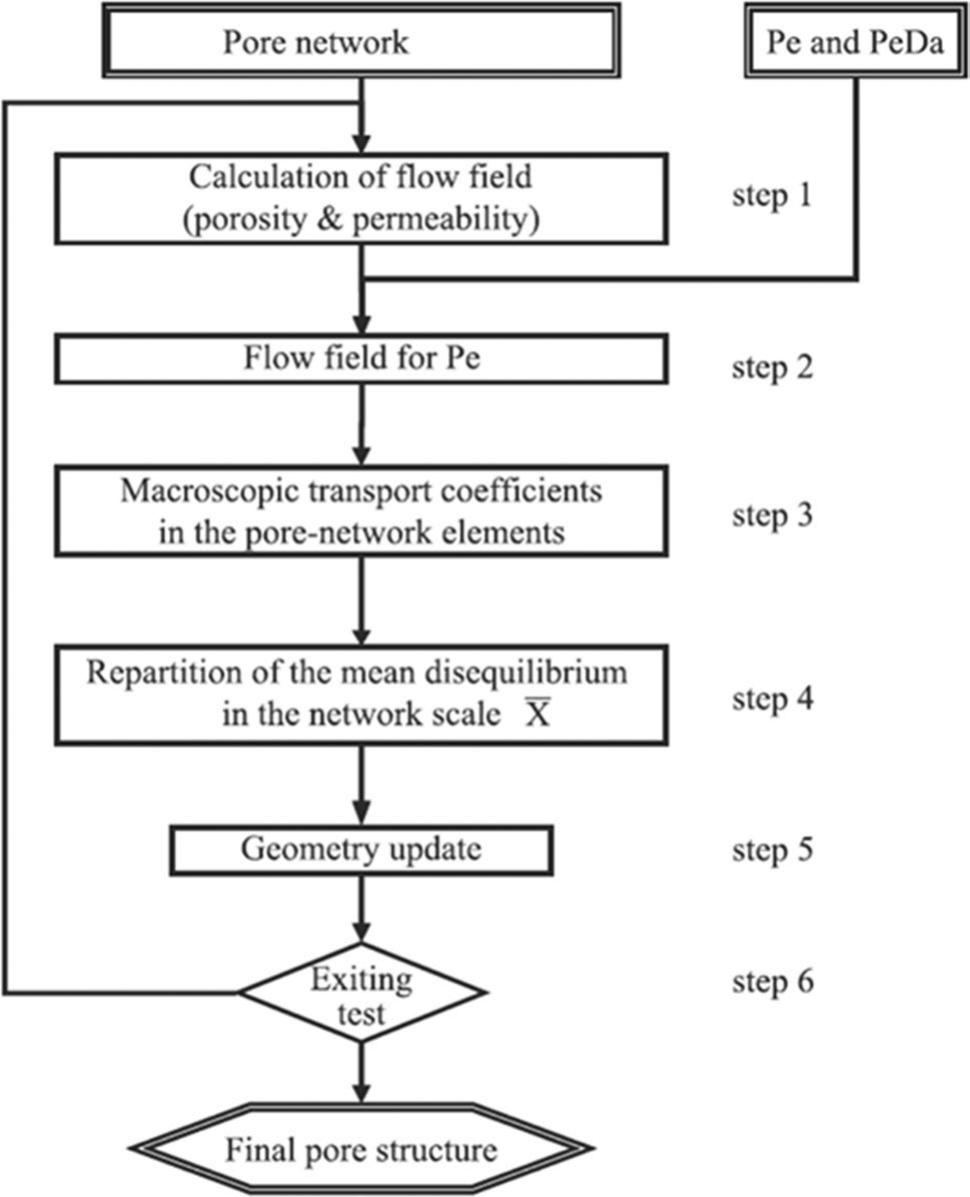
The general scheme of the reactive transport resolution using PNM (Varloteaux et al., 2013)

The computation of how the geometry evolves from the reaction is done through an iterative process based on porosity changes (Fig. 6). The flow field is first determined in step 1 for arbitrary differential pressure for a given initial pore network. Then, with the known mean interstitial velocity, the porosity and permeability of the pore network are determined. In step 2, the velocity field is adjusted through a linear correction applied to the differential pressure to the imposed Peclet number, Pe. The pore-scale transport coefficients are then determined for each pore body and throat. In step 4, the mass balance equation is solved over the whole network to specify each pore body’s mean transverse profile parameter; see Varloteaux et al. (2013) for more details. Finally, in step 5, how the geometry evolves is considered. For consistency with the PNM assumptions, the evolution of the wall is averaged over each pore network’s element to retain the original shape of each element. Subsequently, the wall evolution is modified to have a porosity evolution that is controlled and small. Finally, the pore network is updated in an iterative process from steps 1 to 5 until convergence is reached as the geometry of the network becomes compatible with PNM.

**Fig. 7:**
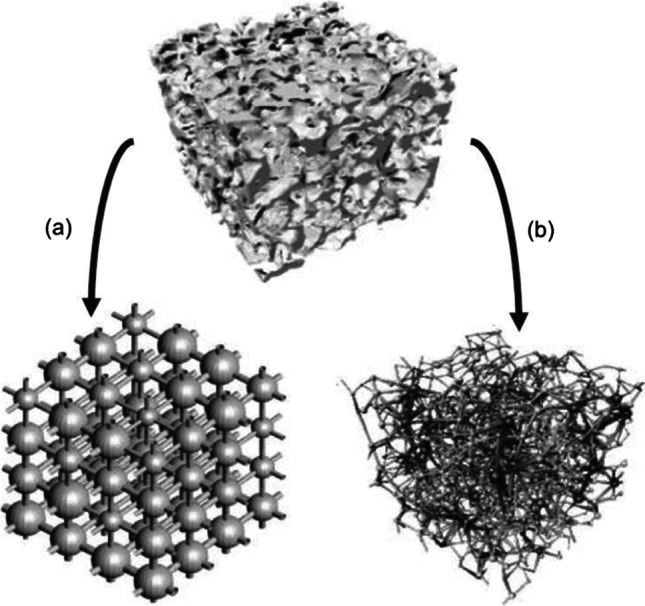
Pore network models (a) reconstructed with a regular lattice to reproduce the real petrophysical properties in the porous media (Bekri and Vizika, 2006), and (b) extracted from microtomography (Youssef et al., 2007)

Constructing a PNM, representing a porous medium, is generally done in three ways: statistical reconstruction, grain-based method and direct mapping model. In the statistical reconstruction technique, the construction of the 3D images can be achieved with information extracted from 2D pore space images. Then the 3D images of the actual pore space can be reconstructed with the knowledge of the pore’s geometrical properties using the truncated Gaussian random field method (Adler and Thovert, 1998). The structure of the pore network can be regular or an irregular 3D lattice Fig. 7. For example, Fig. 7a shows a pore with a cubic lattice structure, with each pore body connected to six pore throats, while Fig. 7b is an extracted image from microtomography. Thus, the coordination number is six, and there is a constant ratio between the pore body and the throat diameter. The diameters of the pore throat are generated randomly using a defined probability density function, and the coordination number and aspect ratio can be varied.

When constructing a representative pore network of a porous medium, the selected probability density function should be able to reproduce geometrical properties required to replicate the medium’s topology parameters, such as permeability and porosity. Bekri and Vizika (2006) recommended that the formation factor and the capillary pressure estimated through this modelling effort can be close in value to the actual porous media being considered since they are sensitive to its structure. This approach is possible if the pore networks can be directly constructed from a 3D image. If the experimental data comes from non-imaging techniques such as mercury intrusion porosimetry and gas adsorption, where the characteristics of the pore space are not available, the regular pore network construction technique is appropriate (Xiong et al., 2016). The reader can check the works of Ioannidis and Chatzis (2000), Adler and Thovert (1998), Roberts and Torquato (1999), Okabe and Blunt (2004) and Yeong and Torquato (1998) for more details about this construction approach.

The grain-based model was introduced by Bryant and Blunt (1992), Bryant et al. (1993a) and Bryant et al. (1993b). The model is based on random close packing of equally sized spheres, with equivalent networks having four or fewer coordination numbers. In the modelling approach, the uniform swelling of the spheres and allowance for overlapping represents diagenesis. In contrast, the movement of the spheres’ centres closer to each other in the vertical direction and allowing for overlap of the spheres represent compaction. This approach can predict the properties of cemented quartz sandstones and water-wet sand packs, such as the capillary pressure, absolute and relative permeabilities and electrical and elastic properties. However, the main disadvantage of this method is that it is primarily applicable to porous media with spherical grains of the same size, But Bakke and Øren (1997), Oren et al. (1998), Lerdahl et al. (2000) and Øren and Bakke (2002) extended the reconstruction method to simulate the packing of spheres with different sizes. In the same vein, attempts have also been made to integrate the extracted networks from images, at specific length scales, into a single two-scale network (Bultreys et al., 2015; Jiang et al., 2013; Mehmani and Prodanović, 2014).

The direct mapping of the real image of the pore networks will often yield an irregular lattice, which allows for verifying the imposed physical assumptions for the simulation of fluid flow. The results of the flow simulations can be compared with a 4D imaging of mass transport in the medium. In this model, medial axis and maximal ball algorithms are used for constructing the irregular lattice model. For more details, read the paper by Dobson et al. (2016).

#### Macroscale approaches

The macroscopic scale approaches assume the porous medium to be an averaged continuum. And in most of the studies on upscaling effective models that show the relationship between the pore-scale characteristics of the medium and/or other coupled processes to the upscaled equivalents are derived (Heße et al., 2009). Some of the upscaling approaches used included volume averaging, method of moments, homogenisation through multiscale expansions and thermodynamically constrained averaging (Battiato et al., 2009). Hence, the fluctuations in the means of governing variables are often disregarded. Though these approaches establish links between physicochemical processes on different scales when the governing assumptions hold, they cannot validate these assumptions or provide the regions of a computational domain where the continuum model fails.

**Fig. 8:**
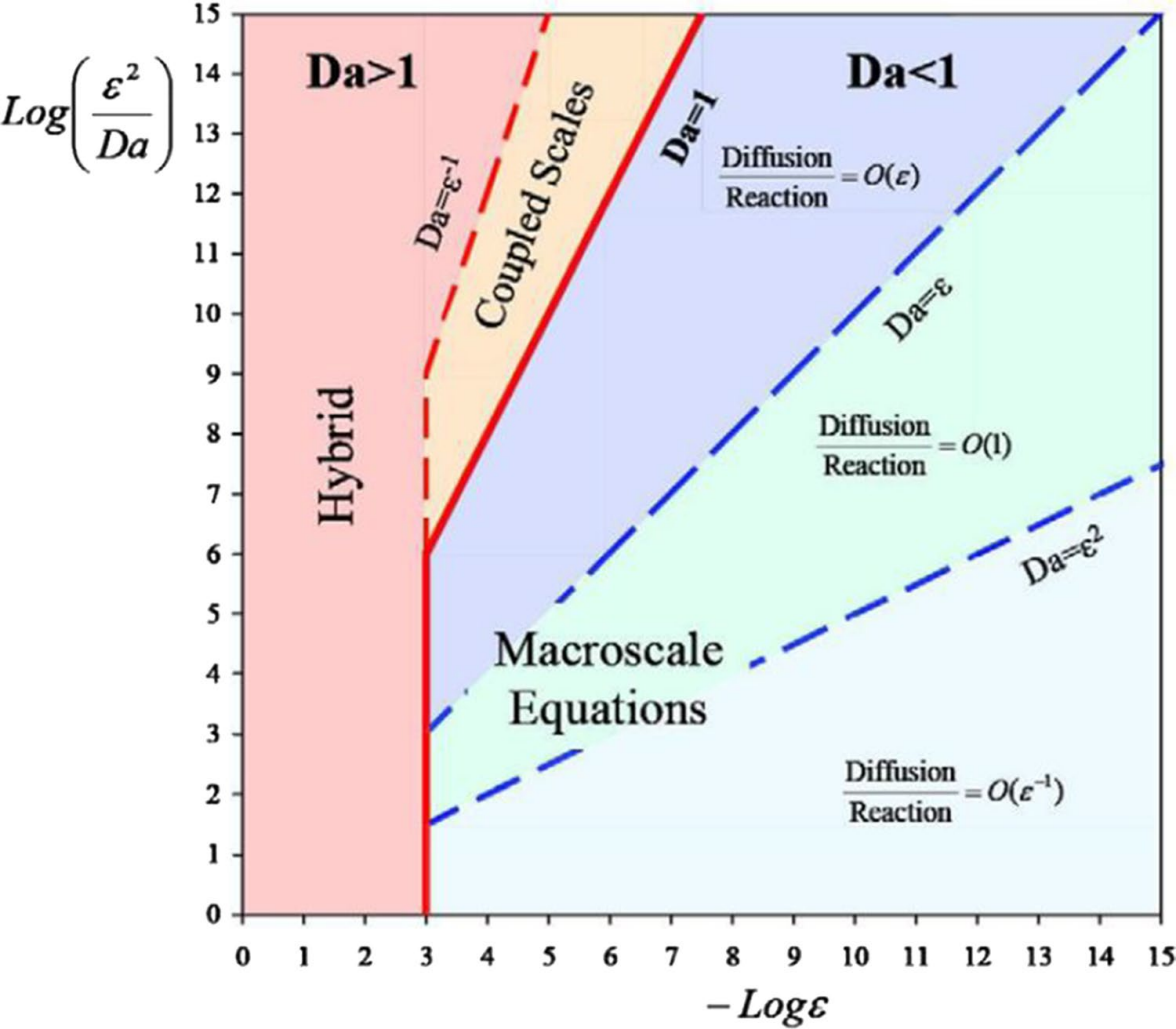
A phase diagram showing the range of applicability of macroscopic models for a reaction–diffusion system in terms of the Da number. The macroscopic models are applicable in the blue region. In the red and orange regions, macroscale and microscale problems (Battiato et al., 2009)

Battiato et al. (2009) used the volume averaging method to upscale mixing-induced precipitation in identifying sufficient conditions for the continuum-based reaction–diffusion equations. In the analysis, they presented a phase diagram that showed the range at which the macroscopic models are applicable. The phase diagram (Fig. 8) showed that highly localised processes in porous media, including mixing-induced precipitation, are not worth using macroscopic models. This is because using such reaction–diffusion equations relies on approximations whose accuracy cannot be determined a priori. The upscaled equations are as follows:

21 $$\begin{array}{cc}\phi \frac{\partial {\langle {c}_{i}\rangle }^{l}}{\partial t}=\frac{{\varepsilon }^{2}}{Da}\nabla .\left(\phi {D}_{eff}.\nabla {\langle {c}_{i}\rangle }^{l}\right)-\phi {\langle {c}_{1}\rangle }^{l}{\langle {c}_{2}\rangle }^{l}+\phi K{\langle {c}_{3}\rangle }^{l}& \left(i=\mathrm{1,2}\right)\end{array}$$

22 $$\phi q\frac{\partial {\langle {c}_{3}\rangle }^{l}}{\partial t}=\frac{q{\varepsilon }^{2}}{Da}\nabla .\left(\phi {D}_{eff}.\nabla {\langle {c}_{3}\rangle }^{l}\right)+\phi {\langle {c}_{1}\rangle }^{l}{\langle {c}_{2}\rangle }^{l}-\phi K{\langle {c}_{3}\rangle }^{l}-q{a}_{v}{l}_{l}\frac{D{a}_{ls}}{Da}\left({\langle {c}_{3}\rangle }^{l}-1\right)$$

where $\phi$ is porosity,$q$ is Darcy flow rate, ${a}_{v}={|A}_{ls}|/|\mho |$ where ${A}_{ls}=\mho \cap {V}_{1}$ and ${V}_{1}\in \mho$, ${l}_{l}$ is the pore-geometry length scale, $\varepsilon ={l}_{1}/{L}_{c}$ with ${L}_{c}$ being the macroscopic length scale associated with$\langle c\rangle =\phi {\langle c\rangle }^{l}$, ${D}_{eff}$ is the effective diffusivity tensor, $K$ is a dimensionless quantity, ${Da}_{ls}$ is the Damkohler number for precipitation or dissolution process and $\mho$ is size of the averaging volume.

Conversely, in upscaling the pore-scale construction of advection and diffusion equations, where the reaction equations are formulated such that they are placed in the boundary conditions of the fluid–solid interface, Battiato and Tartakovsky (2011) used multiple-scale expansions to ensure the macroscopic description of the process accurately represents the pore-scale processes. They also provided a phase diagram to establish the range of applicability of macroscopic models, using a different set of process and media properties. Still, they arrived at the same conclusions as to the previous work. The homogenised form of the advection–diffusion-reaction equation using this upscaling approach was presented as

23 $$\phi \frac{\partial {\langle c\rangle }_{B}}{\partial t}=\nabla .\left({D}^{*}\nabla {\langle c\rangle }_{B}-Pe{\langle c\rangle }_{B}\langle v\rangle \right)-{\varepsilon }^{-1}\phi Da{K}^{*}\left({\langle c\rangle }_{B}^{a}-1\right)$$

subject to the following conditions.

- $$\varepsilon "1$$
- $$Pe<{\varepsilon }^{-2}$$
- $$Da/Pe<\varepsilon$$
- $$Da<1$$
- $${\langle \chi \rangle }_{\Gamma }\approx {\langle \chi \rangle }_{\mathrm{B}*}$$

the dispersion tensor D* in Eq. (23) was given as

24 $${D}^{*}=\langle D\left(I+{\nabla }_{y}\chi \right)\rangle +\varepsilon Pe\langle \chi k\rangle {\nabla }_{x}{p}_{0}$$

while the closure variable with zero mean is defined as the solution to the local problem:

25 $$\begin{array}{cc}-{\nabla }_{y}.D\left({\nabla }_{y}\chi +I\right)+\varepsilon Pe{v}_{0}{\nabla }_{y}\chi =\varepsilon Pe\left({\langle {v}_{0}\rangle }_{B}-{v}_{0}\right)& ,y\end{array}\in B$$

26 $$\begin{array}{cc}-n.D\left({\nabla }_{y}\chi +I\right)=0& ,y\end{array}\in \Gamma$$

where ${v}_{0}= -{\varvec{k}}{\nabla }_{x}{p}_{0}$, ${p}_{0}$ is pressure, ${\varvec{k}}$ is permeability, $\varepsilon$ is a length-scale parameter, $\chi$ is the closure variable and $I$ is the identity matrix. The constraints listed above are critical in ensuring the separation of scales. The first constraint is a common observation in several practical applications, and the remaining four are dependent on the relative importance of the transport’s diffusive, reactive and advective processes.

##### Solution strategies for macroscale approaches

In recent years, efforts to develop numerical solutions for reactive transport processes have focused on how to couple the reaction and transport terms (Yeh and Tripathi, 1991). In the effort to develop such algorithms, several methods have been proposed, dating back to those presented by Rubin (1983). The most rigorous of these algorithms is the attempt to solve the governing equations simultaneously. In this work, three commonly used numerical solution methods are discussed.

A. Finite volume scheme
  Hao et al. (2012b) presented an integrated finite difference solution for a multiphase, multicomponent heat and mass flow and reactive transport in both unsaturated and saturated porous media. Similar model and approach have been implemented before their works or are integrated into the robust codes NUFT (nonisothermal unsaturated–saturated flow and transport), such as the work of Pruess (1991), Nitao (1998), Nitao (2000), Yeh and Tripathi (1991), Xu et al. (1999), Mills et al. (2007), Nichols et al. (1997) and Xu (1998).

One outstanding feature of the NUFT model is its capability to handle the disappearance and appearance of any of the three fluid phases that may occur during evaporation or condensation or immiscible fluid displacement processes. To approximate flow in fractured porous media effectively, NUFT considered using the effective continuum model, which has been described extensively earlier, along with the dual porosity model. The dual porosity/permeability model describes the fracture and matrix systems as two separate overlapping continua, where multiphase flow and heat transfer equations are conceptually addressed in both. In addition, each continuum will have a set of its mass and energy balance equations. For more details on this approach and other relevant equations, the reader is advised to check the following references (Hao et al., 2012b; Buscheck et al., 2003, 2006, 2009).

The finite volume method was applied to discretise the partial differential equations, governing each sub-model’s mass and energy balance equations, and an implicit scheme for the time integration because of its numerical stability. At each time step, the nonlinear system of equations, which results from the discretisation, is solved using the Newton–Raphson algorithm. Each nonlinear iteration step requires solving a set of linear algebraic equations. Therefore, multiple linear solvers and decomposition options, including Gauss elimination preconditioned conjugate method, block Gauss–Seidel preconditioning and incomplete block LU (lower–upper) decomposition were implemented in NUFT. In addition, the numerical stability, robustness and accuracy of the algorithm were further enhanced through some special numerical techniques, including primary variable switching, basis-species switching, artificial diffusion technique and flux-correction methods to reduce numerical dispersion.

The critical issue in numerical simulations of reactive transport is the coupling of the transport and reaction models accurately and efficiently. Generally, most available numerical algorithms proceed into forms: global implicit and operator splitting methods. The operator splitting method separates the transport and chemical models and then sequentially solves them. The representative numerical implementations for this method include sequential iterative and sequential non-iterative approaches. The iterative approach includes iterations for convergence between two solution steps, which requires more CPU (central processing unit) time but has more accurate solutions than the non-iterative method. In the global implicit method, there is a full coupling between the transport and chemical reaction equations, and they are solved simultaneously. Though the computation cost of the global implicit method is high, it enables effective coupling of physical and chemical processes without any inconsistency.

The coupling between the two models is often done with a time-marching sequential solution procedure to reduce the high computation cost in simulating flow and reactive transport processes. This approach avoids the computation of a large Jacobian matrix present in a fully coupled flow and transport procedure. Given a time step, the flow model’s mass and energy balance equations are solved first, and the flow variables are then calculated and incorporated into the reactive transport equation. After that, the reactive transport simulation is then performed using the same flow variables for the time step. Because the chemical reactions time scale is often smaller than flow processes, multiple time steps are usually required for reactive transport calculations to reach the given flow time step. At the end of the computation, porosity and permeability changes caused by the chemical reactions are then updated in the flow equation for the next time step. It should be noted that this same algorithm can be applied to the finite element method as well.

B. Mixed finite element method
  Arbogast et al. (1996) presented a flow and reactive transport simulator called PARSIM (Parallel Simulator). In this work, the simulation of the transport and reactions of dissolved chemical species in the groundwater was done. The time splitting method was used for the advection, diffusion and reaction coupled equations. The method of characteristics and the higher-order Godunov method were provided as options to treat the advection problem. In contrast, the mixed finite element method was used to discretise the diffusion equation. Reactions were handled separately as a differential–algebraic system of equations. The Godunov scheme is applicable when the reactive time steps are of the order of a CFL (Courant Friedrichs Lewy) time step. The Godunov mixed finite element scheme and the characteristic-mixed method have also been implemented for other advection or diffusion problems by Arbogast et al. (1994), Arbogast and Wheeler (1995), Dawson (1995) and Dawson, (1993).
C. Upscaled smoothed particle hydrodynamic method
  Mesh-free smoothed particle hydrodynamics approach was used by Battiato et al. (2009), Tartakovsky et al. (2007a) and Tartakovsky et al. (2007b) to solve the upscaled advection–diffusion-reaction equations of *M* species. The macroscopic quantities were computed from the microscale simulations by averaging them over a representative volume, whose characteristic dimension is such that the characteristic radius is far greater than the pore-geometry length scale. The representative volume was defined as the averaging volume with the minimum radius beyond which the porosity remains invariant with increasing averaging volume. The intrinsic average solute concentration was determined as
  27 $$\begin{array}{cc}{\langle {c}_{1}\rangle }^{l}\left(x\right)=& \frac{1}{N\left(x\right)}\sum\limits_{b\in V}{c}_{1}\left({y}_{b}\right){W}_{x,{r}_{0}}\left({y}_{b}\right)\end{array}$$

where *N* is the number of liquid SPH particles contained in the representative volume is the position vector of particle $b$, and ${W}_{x,{r}_{0}}\left({y}_{b}\right)$ is a weighting function**.**

#### Hybrid approach

Macroscopic models for flow and reactions in porous media sometimes fail to describe observed phenomena in experiments. At the same time, the corresponding pore-scale approaches can accurately capture the observed events, although they may be computationally expensive. Most multiscale models that seek to couple both upscale and microscopic approaches require empirical closure relations about the behaviour of the quantities at pore- and continuum scales (Christie, 1996, Efendiev and Durlofsky, 2003; Langlo and Espedal, 1994). On the other hand, Battiato et al. (2011) proposed a general framework for the iterative hybrid numerical method that couples the microscopic and macroscopic scales without any empirical closure. The formulation assumes the exchanged fluxes at the internal boundaries between the microscopic and macroscopic scale domains are unknown and permits iteratively determining the necessary boundary conditions at the microscopic scale to guarantee flux continuity.

One of the benefits of the hybrid simulation scheme is that it provides significant speed-up in simulations where pore-scale simulations are necessary locally in the computational domain. This is possible because of the low cost of the continuum scale simulations, which is applied for most of the computational domain. Furthermore, as noted by Alexander et al. (2002), choosing a hybrid algorithm provides a great benefit when the interfacial region of interest is very small compared to the entire computational domain. The hybrid formulation is applicable for Darcy flow in the medium (Battiato et al., 2009, 2011).

The hybrid pore-scale/continuum-scale algorithm developed by Battiato et al. (2011) has three dependent variables that satisfy a system of coupled partial differential equations:

28 $$\phi \frac{\partial \mathrm{c}}{\partial t}+\phi \nabla .\left(V\mathrm{c}\right)=\nabla .\left({{\varvec{D}}}^{\boldsymbol{*}}\nabla \mathrm{c}\right)-{K}_{e}\mathrm{c} x\in {\Omega }^{T} t>0$$

29 $$\begin{array}{cc}\phi \frac{\partial \mathrm{c}}{\partial t}=\frac{1}{\phi \Vert {\Omega }_{p}\Vert }{\int }_{{\Gamma }_{ll}}{q}_{n}dx-\frac{1}{\phi \Vert {\Omega }_{p}\Vert }{\int }_{{\Gamma }_{sl}}\mathcal{K} cdx& x={x}^{*}\end{array} t>0$$

30 $$\begin{array}{cc}\frac{\partial c}{\partial t}+\nabla .\left(vc\right)={{\varvec{D}}}^{\boldsymbol{*}}{\nabla }^{2}c& x\in {\Omega }_{p} t>0\end{array}$$

31 $$\begin{array}{cc}-n.{{\varvec{D}}}^{\boldsymbol{*}}\nabla c=\mathcal{K} c& x\in {\Gamma }_{sl}\end{array} t>0$$

32 $$\begin{array}{cc}n.\left(\mathcal{D}\nabla c-vc\right)={q}_{n}& x\in {\Gamma }_{ll}\end{array} t>0$$

where $c={c}^{^{\prime}}+\overline{c }$ is the pore-scale concentration, $\overline{c }$ being the mean concentration and ${c}^{^{\prime}}$ the fluctuation, $\mathcal{D}$ is the molecular diffusion, ${{\varvec{D}}}^{\boldsymbol{*}}$ is the dispersion tensor, $\mathcal{K}$ is the reaction rate constant for a surface reaction, $n$ is the outward unit normal vector of the solid–fluid interface ${\Gamma }_{sl}^{T}$. ${q}_{n}$ is the flow rate, ${K}_{e}$ is the effective reaction rate, ${\Omega }_{p}\subset\Omega$ is the computational subdomain where pore-scale simulation is required and $t$ is time.

The initial and boundary conditions on the computation domain are also added to these equations. It should be noted that porosity is assumed to be constant in this formulation. In a case where porosity evolves, the equation can easily be modified to account for that, unlike other multiscale approaches that decouple the two descriptions (pore-scale and continuum scale) by using closure assumptions to show the undetermined pore-scale flux ${q}_{n}$ based on its continuum scale correspondence. One form of strategy representing the pore-scale concentration is the sum of its average and the corresponding fluctuations. Other techniques are linearising the concentration function, postulating a numerical or analytical closure for the corresponding fluctuations, and imposing boundary conditions on the interface.

The hybrid strategy employed by Battiato et al. (2011) computes the undetermined pore-scale flux with no assumption of the pore-scale behaviour and without linearising the general reactive term if present. Hence, the pore-scale concentration is obtained from Eq. (29) by solving the transport problem defined in the subdomain. And the boundary condition $n.D\nabla c=\mathcal{H}c$ ($\mathcal{H}$ is the reaction rate constant for a surface reaction) is now represented on the union of all the solid–liquid surfaces contained in the subdomain. While on the fluid–fluid segments, the mass conservation requires that $n.(D\nabla c-{\varvec{v}}c)={q}_{n}$.

## Evolution of continuum-scale parameters

Parameterisation of an evolving porous medium can be challenging, especially in the continuum scale. To capture such a phenomenon, some tools have been proposed. This section presents a review of common methods available in the literature to parameterise evolving parameters for simulations of reactive transport such as porosity, transport parameters and the existing relationships among the rate of flow and transport processes. These processes are discussed at the pore-scale, although with a significant impact at the macroscale.

### Porosity evolution in porous media

As mentioned in the previous section, as the minerals dissolve and/or are precipitated, it can lead to changes in the porosity of the porous media. Estimating the total change in porosity at the macroscale is determined at any point in time, based on the volume fractions of the mineral that make up the grain matrix, as (Appelo and Postma, 2005, Hommel et al., 2018)

33 $$\phi =1-\sum\limits_{i}^{{N}_{m}}{\varphi }_{i}$$

It is worth noting that this equation defines total porosity, while effective porosity is not addressed. In a situation where the mineralogical composition of the media is unknown, and the dissolution of few phases contributes to porosity changes, the equation below can be applied.

34 $$\phi =1-{\varphi }_{NR}\sum\limits_{i}^{{N}_{m,r}}{\varphi }_{i}$$

${\varphi }_{NR}$ is the volume fraction of the non-reactive minerals, and ${N}_{m,r}$ is the number of reactive minerals, and ${\varphi }_{i}$ is the mineral volume fraction of a mineral phase**.** Hence, the change in porosity with time can be determined from

35 $$\frac{d\phi }{dt}=-\sum\limits_{i}^{{N}_{m,r}}\frac{d{\varphi }_{i}}{dt}$$

The effective porosity of the medium can also be updated through a similar procedure. One of the limitations of the previous equation is that it fails to provide any detailed description of the structural evolution of the pore, although it accurately provides a description of the foregoing processes. However, other attempts have developed macroscaled relationships to describe the evolution of the pore structure indirectly.

### Diffusivity and tortuosity as functions of porosity

When there is an absence of the advective fluxes, diffusion dominates the transport of solute. Therefore, for deep geologic repositories, the dominant transport process must be diffusion. And to better understand the degradation process of cementitious materials at the interface of clay and cement in radioactive waste repositories, efforts have been applied using reactive transport modelling (Dauzères et al., 2019, Savage, 2013, Jenni et al., 2017, Gaucher and Blanc, 2006). But it should be noted that the diffusion properties of the media are liable to vary as the porosity and tortuosity evolve because of dissolution and precipitation processes. As defined by Bear (1988), tortuosity is the ratio of the path taking by the solute in water to that followed through the rock.

Several reactive models have been established to describe the correlation between tortuosity and porosity and the direct influence of porosity on diffusion. In these relationships, which are mainly applicable to evolving porous media, the effective diffusion coefficients are determined from porosity, which is easy to measure. One of the relationships often used is Archie’s law which shows the dependence of porosity on the pore diffusion coefficient. The simplest form of the relationship between tortuosity and porosity is

36 $${\tau }_{l}={\phi }^{{n}_{c}}$$

where is ${\tau }_{l}$ tortuosity, $\phi$ is porosity, and ${n}_{c}$ is the cementation factor. Recall that the saturated aqueous phase effective diffusion coefficient is Seigneur et al. (2019):

37 $${D}_{l}^{e}=\phi {\tau }_{l}{D}_{l}^{0}$$

A generic form of relationship for the liquid phase effective diffusion coefficient is presented in this paper in Eq. (44). This relation reduces to the form presented by Seigneur et al. (2019) when $k=2$ and (Oelkers, 2018) when ${D}_{l}^{min}=0$:

38 $${D}_{l}^{e}={\phi }_{e}^{k}{D}_{l}^{0}+{D}_{l}^{min}$$

where ${D}_{l}^{0}$ is the pure water diffusion coefficient, ${D}_{l}^{min}$ is the matrix effective diffusion coefficient, $k$ is an empirical parameter and ${\phi }_{e}$ is the effective porosity, defined as

39 $${\phi }_{e}=\left\{\begin{array}{cc}a{\left(\phi -{\phi }_{crit}\right)}^{\beta }& \phi \ge {\phi }_{crit}\\ 0& \phi <{\phi }_{crit}\end{array}\right.$$

The constant $a$ is a fitting parameter, $\beta$ is the scaling exponent, as defined by Stauffer and Aharony (1985) and Ellis and Wright (2006), and ${\phi }_{crit}$ is a critical porosity**.** Another form of relationship for estimating effective porosity was presented by Tarafdar and Roy (1998) as:

40 $${\phi }_{e}=\varepsilon \phi$$

where $\varepsilon$ is the pore connectivity fraction.

There are other forms of relationships for tortuosity, including an exponential relationship with porosity, which are not covered in this review. For an unsaturated condition, Millington and Quirk (1961) showed that phase saturation is a variable that affects tortuosity. They presented an empirical model of the form:

41 $${\tau }_{\alpha }={S}_{\alpha }^\frac{7}{3}{\phi }^\frac{1}{3}$$

Equation (47) shows that tortuosity has a strong nonlinear relationship with saturation, ${S}_{\alpha }$, which (Millington, 1959) derived for gas diffusion in porous media. However, in most reactive transport simulations, the relation proposed by Millington and Quirk (1961) in modelling the diffusion through the liquid phase is adopted. On the other hand, the application of this model to unsaturated conditions is questionable. A review of several models for unsaturated conditions was presented by Chou et al. (2012) and affirmed the inaccuracy of the Millington-Quirk model.

When applying reactive transport modelling, the deterioration of cementitious materials is enhanced by dissolution reactions, driven by the solute diffusion into these materials. The extent of degradation into these materials is dependent on the cementation factor or the type of feedback law used. This is because the diffusion of calcium into the affected areas strongly impacts the dissolution rates of the primary minerals. In other studies on reactive transport, the extent of the degradation in the material was estimated using either Archie’s law (Georget et al., 2018; De Windt and Badreddine, 2007; Li et al., 2017; Galíndez et al., 2006; Chagneau et al., 2015) or an exponential correlation (Mainguy et al., 2000; Seigneur et al., 2017) for the description of the relationship between evolving porosity and tortuosity variation. But the selection of the cementation factor used in these studies is strongly dependent on the materials being considered. Other relevant contributions on this topic include the works of Maxwell (1881), Petersen (1958) and Tomadakis and Sotirchos (1993).

In most modelling studies, the initial porosity and tortuosity, representing the state and type of the material of interest, are imposed. These empirical relations are then used to compute how the diffusion parameters evolve in the process. In these attempts, curve-fitting of the early time data or independent approaches is used to determine the media’s initial diffusion properties. Knowing that dissolution within the regime controlled by diffusion occurs uniformly in the domain can cause a slight increase in tortuosity. Using this technique has resulted in a close agreement between modelling and experimental data. However, it is often difficult to know the dynamic interactions between precipitation and dissolution processes and the consequent effects on tortuosity and diffusion parameters when both occur simultaneously. Others attempt to tune the empirical factors of the precipitation reaction artificially (Huet et al., 2010; Chagneau et al., 2015; Brunet et al., 2013), modify the parameters defining the reactivity (Jacquemet et al., 2012) or employ other empirical relations (Walsh et al., 2013) to mimic breakthrough curves and degradation extent in materials under carbonation process.

Following these modelling approaches, their results have been consistent with pore-scale simulations. Hence, it suggests that localised precipitation of minerals can significantly affect the paths of diffusion. Consequently, it is evident from the foregoing that the effect of precipitation reactions on diffusion is not easy to capture compared to the impact on dissolution. Also, it can be implied that the reactions of different pore materials to these reactive processes strongly depend on the state of their initial pore structures. In this light, porous media having high initial tortuosity and small pore throats are highly sensitive to actions of precipitation (Seigneur et al., 2017; Brunet et al., 2013; Kutchko et al., 2007; Huber et al., 2014). Furthermore, it suggests that using a specific correlation between tortuosity and porosity for all materials may not be advisable because different materials respond to dissolution and precipitation reactions differently. However, understanding the impacts of these reactions on the diffusivity of materials remains unresolved because of the coupled nature of these reactions.

### Permeability as a function of porosity

The advection-driven transport regime is influenced by the evolution of the permeability of the porous media. In this regime, porosity alteration can affect permeability, and thus, the fluid flow patterns, which can impact the degree of chemical fluctuations that can alter the nature and structure of the medium. Hence, using relationships that can capture the impact of evolving porosity on permeability is often advised. One prominent power law relation is the Kozeny-Carman model (Hommel et al., 2018, Appelo and Postma, 2005), which can simulate permeability evolution affected by the dissolution and precipitation of minerals. Its commonly used form is:

42 $$k={k}_{0}\frac{{\phi }^{3}}{{\left(1-\phi \right)}^{2}}\frac{{\left(1-{\phi }_{0}\right)}^{2}}{{\phi }_{0}^{3}}$$

where ${k}_{0}$ and ${\phi }_{0}$ are the initial permeability and porosity, respectively; other forms of power law models have been used extensively to model permeability evolution (Hommel et al., 2018). In the modelling efforts, capturing the evolution of diffusivity is challenging. Within regions where diffusion dominates, there is a measure of stability in the reaction fronts. On the other hand, within the regimes dominated by advection, the distribution of flow rates is affected by pore-scale heterogeneities, which can cause the fronts to be unstable. Alternatively, it is common to use cubic relations for permeability evolution related to the local fracture aperture. This form of relationship has long been used, dating back to the work of Witherspoon et al. (1980). Oron and Berkowitz (1998) generalised the local cubic law for rough fractures. For more details on this topic, the reader is referred to the work of Deng and Spycher (2019).

Furthermore, as pointed out by Hommel et al. (2018), it is worth noting that generally the use of the simple power law model provides similar accurate permeability predictions as the more complex models. But with increased porosity reduction, there is a great disparity between them and the simple power law model. Hence, it is recommended that the simple power laws be used as default for modelling an evolving medium. While more complex models be used to capture known processes alongside the porosity evolution of the medium.

### Evolution of reactive surface area

Mineral dissolution and precipitation not only result in modification of the transport properties, but they also alter reactivity, causing the reaction rates to be enhanced or decreased. Understanding the evolution of reaction rates is equally important as knowing how the transport parameters evolve. Therefore, it is critical to have an adequate description of the reaction rates to capture concentrations and loadings of the solutes for the long term. Also, having a proper definition of reactions is essential for pH-buffer reactions to capture the interactions with the flow and transport processes. To describe reactivity, the reactive surface area of the minerals is often used. As observed by Emmanuel and Berkowitz (2007), Liu et al. (2008), Landrot et al. (2012), Peters (2009), Lai and Krevor (2014) and Deng et al. (2018), several factors affect the reactive surface area, including grain size, exposure of the mineral to pore fluid, degree of occlusion, surface roughness and etch pits. However, the presence of multiple phases causes the surface fraction to differ significantly from its fractional volume (Lai and Krevor, 2014). Furthermore, the flow state can increase the formation of more reactive zones, consequently contributing to alterations of reactive areas from the geometric surface area; thereby, the effective reactive surface area is controlled (Jung and Navarre-Sitchler, 2018).

Different mathematical modelling attempts have been made to describe how the surface evolves. One of the simplest models assumes the dissolution of a spherical grain is uniform, and the surface area has an inverse proportionality relationship to its radius. Based on this relationship, Steefel and Lichtner (1998) proposed that the exponent of the power law relationship between surface area evolution $(S)$ and porosity $(\phi )$ be two-thirds.

43 $$S={S}_{0}{\left(\frac{\phi }{{\phi }_{0}}\right)}^\frac{2}{3}$$

where ${S}_{0}$ and ${\phi }_{0}$ define the surface area and the porosity, respectively. This same relationship has been used on the continuum scale, although it was derived based on a single-grain assumption. In some instances, the reduction in reactivity and surface area as dissolution proceeds is intuitively assumed; unfortunately, this assumption fails in some cases. The reactive surface area can also be increased through the dissolution processes, as observed by Noiriel et al. (2009). He noticed that dissolution reactions could have the same effect of etch pits on the mineral surface area, thus increasing the reactive surface area. Another power law relationship for describing the evolution of the surface area with porosity was presented by Luquot and Gouze (2009):

44 $$S={S}_{0}{\left(\frac{\phi }{{\phi }_{0}}\right)}^{-w}$$

where $w$ is a fitting parameter. The reader is also advised to check the work of Noiriel et al., (2012), Molins et al. (2014), Molins et al. (2019), Ritchie (1994), Wunderly et al. (1996), Lefebvre et al. (2001), Mayer et al. (2002), Steefel and Lichtner (1994), Steefel and Lichtner (1998), Deng et al. (2016), Dentz et al. (2011a), Luhmann et al. (2014) and Appelo and Postma (2005) for more details.

The reactive surface area of the primary minerals can also be affected by the secondary minerals precipitated, which can cause the passivation to the reaction surface. Capturing this passivation can be challenging because of the complexity in the surface morphology of the secondary phases. Conceptually, it can be viewed that the reactivity of the phases of the primary minerals can be linked to the fractional volume of the secondary minerals. Jeen et al. (2007) presented an exponential decay function to describe the evolution of the reactive surface of iron present in a permeable reactive barrier:

45 $$S={S}_{0}\mathit{exp}\left(-a{\varphi }_{p}\right)$$

where ${\varphi }_{p}$ represents the total volume fraction of precipitated secondary carbonate minerals, $a$ is a fitting parameter, and ${S}_{0}$ is the initial surface area**.** Harrison et al. (2016), Daval et al. (2009) and Noiriel et al. (2012) are other relevant studies on the evolution of reactive surface area during mineral precipitation that the reader can check for more details.

### Thermal, hydrological, mechanical and chemical (THMC) coupled model

This is the most recent and developed model used to assess some subsurface applications such as oil/gas production, geothermal reservoir and repository of nuclear waste, where heat and fluid transfer take an essential role in the process. The original versions of this model were THC and THM, which both imitate the interaction between two critical couplings processes such as heat transfer, hydraulic fluid flow, mechanical deformation and chemical reactions in geological repositories. The “coupling” term indicates that it is a two-way process of interaction where one process will affect and initiate other processes (Lanru and Xiating). Some examples of those coupling are TM (temperature effect on mechanical deformation), TC (temperature effect on chemical reactions), HC (fluid transport effect on chemical reactions) and HM (fluid pressure effect on mechanical deformation) (Zheng et al., 2017). A full THMC coupled model was developed to illustrate the interaction between the mechanical and chemical processes over THM and THC models. The model is based on a numerical analysis which allows the imitation of complex coupling interactions.

In the engineered barrier system, clay minerals are generally used as a buffer because of their high retention capacity, low thermal conductivity, along with low permeability, and diffusion coefficient. Through time, the performance of the clay repository is affected by complex thermal, hydrological, mechanical and chemical (THMC) processes (Zheng et al., 2012). The clay will be subjected to several changes in temperature and hydration level as heat is released from the radioactive waste through the radionuclide decay phenomenon and water filtration from neighbour rocks. In addition, chemical reactions, formation damage and multiphase flow might also take place and disturb the integrity of the repository. To establish a mathematical formulation for the full THMC model, balance equations were developed based on the THM model and then a chemical formulation was added to it.

#### THM theoretical formulation

This model is done by Olivella et al. (1994), and it is based on the theoretical model of multiphase and species, which describes the mechanics of fracture geomaterials. The model considers the porous medium as solid, liquid and gas phases. The precipitated minerals represent the solid phase; the liquid phase contains water and dissolved chemicals, while dry air and water vapour make up the gas phase (Gens et al., 2010). Two coupling processes are established in this model, the thermo-elasticity process (T-M), which imitates thermal stress and expansion of solids, and the poroelasticity process (H-M), which imitates the deformation of porous media (Lanru and Xiating). Hook’s, Fourier’s and Darcy’s laws were used to define elasticity deformation, heat transfer and fluid flow, respectively. Subsequently, balance equations are made as below:

46 $$\frac{\partial }{\partial t}\left[{\rho }_{s}\left(1-\phi \right)\right]+\nabla .{j}_{s}=0$$

47 $$\frac{\partial }{\partial t}\left({\rho }_{l}\phi \right)+\nabla .{j}_{l}={f}^{w}$$

48 $$\frac{\partial }{\partial t}\left[{{E}_{s}\rho }_{s}\left(1-\phi \right)+{E}_{l}{\rho }_{l}{S}_{l}\phi \right]+\nabla .\left({i}_{c}+{j}_{{E}_{s}+}{j}_{{E}_{l}}\right)={f}^{Q}$$

49 $$\nabla .\sigma +b=0$$

where $\phi$ is porosity, ${\rho }_{s}$ is solid density, ${\rho }_{l}$ is liquid density, ${j}_{i}(i=s,l)$ total mass flux, $\sigma$ is stress tensor, $b$ is body force vector, ${E}_{i}(i=s,l)$ is specific internal energy, ${j}_{{E}_{i}}(i=s,l)$ is energy flux due to mass motion, and ${f}^{w}$ and ${f}^{Q}$ are sink/source terms for water mass and energy, respectively. ${i}_{c}$ is conductive heat flux (Gens et al., 2010).

#### Chemical formulation

This model is used to describe the equation of chemical species reactive transport, and it is formulated based on the mass continuity of those chemicals in the porous medium (Gens et al., 2010; Olivella et al., 1994). The chemical model will account for the chemical reactions of cation exchange, acid/base, aqueous complexation, surface complexation and minerals dissolution/precipitation (Zheng et al., 2008, 2012)

50 $$\frac{\partial }{\partial t}\left(\phi {S}_{l}{\rho }_{l}{U}_{j}\right)+\nabla .\left[{\rho }_{l}{\left(Ua\right)}_{j}{q}_{l}-{D}_{l}\nabla {\left(Ua\right)}_{j}+\phi {S}_{l}{\rho }_{l}{U}_{j}\dot{u}\right]+\sum_{m=1}^{{N}_{m}}{v}_{jm}{r}_{m}=0 \left(j=\mathrm{1,2},\dots ,{N}_{c}\right)$$

51 $${U}_{j}={C}_{j}+\sum_{i=1}^{{N}_{x}}{v}_{ij}{X}_{i} \left(i=\mathrm{1,2},\dots ,{N}_{c}\right)$$

52 $${\left(Ua\right)}_{j}={\lambda }_{j}{C}_{j}+\sum_{i=1}^{{N}_{x}}{v}_{ij}{\lambda }_{i}{X}_{i} \left(i=\mathrm{1,2},\dots ,{N}_{c}\right)$$

where ${U}_{j}$ is total analytical concentration, ${(Ua)}_{j}$ is total aqueous concentration of the primary species *j*, ${C}_{j}$ and ${X}_{i}$ are concentrations of the primary and secondary species, respectively. ${\lambda }_{j}$ and ${\lambda }_{i}$ are mobility of primary and secondary species, respectively. ${r}_{m}$ is rate of precipitation or dissolution of mineral m under kinetics conditions. ${N}_{m}$ is number of minerals under kinetics conditions, ${v}_{jm}$ is the mole number of primary species *j* in a mole of mineral *m*. ${N}_{c}$ is a number of primary species, ${N}_{x}$ is a number of secondary species (Gens et al., 2010).

## Speciation methods

Speciation is the process of identifying, estimating quantitatively, or describing the various species or phases present in a material. Several attempts and reviews have been made on speciation methods for radionuclides and metals in sediments and surface waters (Markich, 2002, Von Gunten and Beneš, 1995; Tessier and Turner, 1995; Salbu et al., 2001). Generally, there are two main approaches or methods in speciation, analytical and computational methods. In analytical methods, the nature of the material or medium to be investigated and its phases are very important. But it is worth noting that no single method will provide indisputable information about the material. Often, more than two or more techniques are combined using a speciation scheme. The choice of a speciation scheme is strongly dependent on the nature of the medium. The different analytical techniques can be grouped into two sub-methods—invasive and non-invasive. In invasive techniques, the samples require to be pre-treated, while in non-invasive techniques, there is no need for pre-treatment. The reader can refer to Markich (2002) for a comprehensive list of different analytical methods.

Because analytical methods cannot adequately determine uranium speciation in natural surface waters, computational methods are often used. And most of the information on its speciation in natural waters are determined through thermodynamic modelling. Two separate approaches are usually used: the equilibrium constant and Gibbs free energy methods, although they are both subject to mass balance and chemical equilibrium conditions. In the equilibrium constant method, the expressions of the mass action are incorporated into the mass balance equations, which results in a system of nonlinear equations. On the other hand, the Gibbs free energy method involves transforming the governing variables using their thermodynamic relations. For more details on the classification, the reader can refer to Smith (1982) Van Zeggeren and Storey, (2011), and Zeleznik and Gordon (1968).

There are several geochemical solvers based on the equilibrium constant approach, including WATEQ by Truesdell and Jones (1974), MINTEQA2 by Allison et al. (1991), CHESS by Van der Lee and De Windt (2002) and PHREEQC by Parkhurst and Appelo (1999), Parkhurst and Appelo (2013), and Appelo and Postma (2005). Although the determination of the stable equilibrium phase is challenging and computationally expensive in these codes, this issue has since been resolved using heuristic techniques (Bethke, 2022). However, there are other issues associated with some of these solvers, such as using an incomplete Newton scheme. The reader is advised to check the works of Leal et al. (2013) and Leal et al. (2014) for more details.

Similarly, there are geochemical codes based on the Gibbs free energy method, including CHEMSAGE by Eriksson and Hack (1990), THERIAC by de Capitani and Brown (1987) and GEM-Selektor by Karpov et al. (1997), Karpov et al. (2001), Karpov et al. (2002), and Kulik et al. (2013). Unfortunately, some of these packages also have limitations. For instance, in CHEMSAGE, the code cannot converge at a quadratic rate in the neighbourhood of the solution, GEM-Selektor does not use logarithmic barrier functions often used by other nonlinear programming software.

## Mixing in reactive transport modelling

Mixing is a form of potential interaction between pairs of molecules of reactants in a mixture, and it is affected by two main mechanisms. The first of these mechanisms is advective transport. Without molecular diffusion, mixing depends on the initial and boundary conditions on which the reacting species enter the flow domain. Also, it depends on the configurations of the streamline that drive the reactants very close to enable reaction. The other mechanism is diffusion, which helps transfer reactants between streamlines, furthering interactions among the molecules.

In the macroscale reactive transport modelling approach, it is assumed that there are well-mixed conditions at the pore scale, and changes in the concentration of the species based on chemical reactions occur on time scales greater than the mixing time. This implies that the diffusion time is faster than the reaction.

Incomplete small-scale mixing can significantly impact both heterogeneous and homogeneous reaction rates. Meile and Tuncay (2006) studied its impact and whether the continuum description can still be applied. Li et al. (2008) similarly presented numerical and experimental studies challenging the necessity of the well-mixed condition for the Darcy-scale reactive transport modelling approach.

From the foregoing, it is evident that correctly quantifying the mixing and spreading of the reactants is very important for reactive transport modelling in heterogeneous media. In homogeneous media, mixing and spreading are the same but different in heterogeneous media. The heterogeneities of the porous medium and spatial fluctuations of the flow field can distort the plume of the solute. On a time scale smaller than the time for mass transfer, these mechanisms will increase the spread of the solute but not mixing (Kitanidis, 1994). Although these mechanisms are coupled, they are often separated in heterogeneous media. For instance, as spread leads to the spatial concentration gradient, it thus enhances diffusion and local dispersion, further enhancing mixing.

Dentz et al. (2011b) mentioned that mixing controls the chances of reactants meeting in a flowing fluid. In a macroscopic description, the transport equation for two aqueous species is

53 $$\phi \frac{\partial {c}_{j}\left(x,t\right)}{\partial t}+\nabla .\left(q\left(x\right)-D\nabla \right){c}_{j}\left(x,t\right)=r\left(x,t\right)$$

and reaction rate $r(x,t)$ is

54 $$r\left(x,t\right)={k}_{r}\left(1-\Omega \right)$$

55 $$\Omega =\frac{{c}_{1}\left(x,t\right){c}_{2}\left(x,t\right)}{K}$$

where $\phi$ is porosity, ${\varvec{q}}$ is specific discharge, ${\varvec{D}}$ is the dispersion tensor (the reader is referred to the work of Dentz et al. (2011b) for more description of the dispersion coefficient), and ${k}_{r}$ is the kinetic rate constant.

For the continuum description here to hold, the two reactants must mix at a very fast rate compared to the reaction time scale. For such to happen requires that the microscopic Damkohler number ${Da}_{mic}={\tau }_{D}^{mic}/{\tau }_{r}\ll 1$ with ${\tau }_{D}^{mic}={l}_{p}^{2}/{D}_{mic}$; note that ${\tau }_{r}$ is the reaction time scale. The implication is that the reaction is very slow compared to the transport on a microscopic scale. They assumed that the time for changes in concentration due to bulk transport, ${\tau }_{t},$ is greater than the reaction time, thus requiring that $Da={\tau }_{t}/{\tau }_{r}\gg 1$. When the Damkohler number is large, the system approaches equilibrium, which implies saturation $\Omega =1$. This means the mass action law for the bulk species concentrations, i.e. ${c}_{1}\left(\mathrm{x},\mathrm{t}\right){c}_{2}\left(\mathrm{x},\mathrm{t}\right)=\mathrm{K}$. In this limit, the reaction involving the two reactants is controlled by mixing. Kitanidis (1994) characterised mixing in terms of entropy, defining the entropy of a continuous distribution $c(x,t)$ as

56 $$H\left(t\right)=-\int c\left({\varvec{x}},t\right)lnc\left({\varvec{x}},t\right)$$

This quantifies the degree of disorderliness in the transport process, while $E\left(t\right)=\mathrm{exp}[-H\left(t\right)]$ is called the dilution index. He showed that the rate of change of dilution in the system with time is

57 $$\frac{d lnE\left(t\right)}{dt}=-\int \nabla lnc\left({\varvec{x}},t\right)D\nabla lnc\left({\varvec{x}},t\right) c\left({\varvec{x}},t\right)$$

Dentz et al. (2011b) identified this function as a measure of mixing of the system. The implication is that in the absence of diffusion and dispersion, the entropy corresponds to the entropy of the initial system. Hence, in the absence of dispersion, there is no mixing, and the system’s entropy is not increasing. There is a decrease in the maximum concentration with mixing, and the concentration gradient becomes gentle. Thus, leading to decreased variability in concentration in the system.

De Simoni et al. (2007) proposed a mixing ration-based formulation for multicomponent reactive transport. The goal of the proposed strategy is to evaluate the concentrations of the solute and reaction rates when mixing drives the equilibrium aqueous reactions and precipitation or dissolution of minerals. Their approach decouples the solute transport and the speciation problems to ensure mixing ratios are estimated by solving the conservative transport equation and then using it in the general speciation models to obtain the concentrations of the reactants. The four-step strategy includes (1) evaluation of the mixing ratios, (2) evaluation of components, (3) speciation calculations and (4) calculation of reaction rates.

For further reading on mixing in reactive transport modelling, the reader is referred to De Simoni et al. (2007), Tartakovsky et al. (2009), Dentz et al. (2011b), Soler-Sagarra et al. (2022), and Appelo and Postma (2005), and other cited references by these authors for more details.

## Summaries

The homogeneous and heterogeneous reactions of biochemical species in dissolution in the liquid phase are partly responsible for making complicated reactive transport simulations in porous media. Using Pe and Da, the relative significance of advection, reactions and molecular diffusion can be quantified. Establishing sufficient conditions where macroscopic scale equations for coupled advection, dispersion and reaction process adequately provide a description of the processes at pore-scale has been done by using multiple-scale expansions in upscaling the pore-scale equations to the macroscopic scale while entering the reactions through a boundary condition on the interfaces between the fluid and solid phases. The volume averaging method has also been used to achieve the same results observed in the multiple-scale expansion approach. The phase diagrams developed through these techniques revealed that these transport processes occurring at the pore-scale are quite difficult to describe by macroscopic approaches. On these bases, the governing assumptions and approximations on which the macroscopic models are based cannot be ascertained a priori. In transport regimes, where continuum equations break down, the use of nonlocal or hybrid pore-scale/continuum-scale models is valid. These models present rigorous frameworks compared to the traditional upscaled models that are based on developing closure approximations.

Despite the inherent challenges of evolving relevant parameters that describe the properties of the evolving media in the macroscale approach, the quantitative analysis of the reactive transport has been successful. In addition, the use of hybrid approaches has provided the necessary tools to further understand reactive transport processes in porous media. But it is worth noting that the empirical correlations are not for physically representing the exact pore-scale processes. Hence, these relationships would not lead to a comprehensive process-based assessment of the experimental observations. Nevertheless, there is sufficient understanding to model specific processes through these empirical correlations.

It should be noted that PNM can be applied to any length scale where the pore space structure has been experimentally observed and analysed. The key elements of the PNM approach, sites and bonds can be abstract but are relatable to the measurable features in various ways, depending on the available information. It is possible to extract individual pore networks at each length scale and integrate it into a single multiscale network by characterising the cross-scale link structure between these networks. In the upscaling of reactive transport processes from microscopic to macroscopic scales, pore network models have proven to be an effective research tool. Despite the attractiveness and successful applications of PNMs, successfully simulated transport processes demand adequate representation of the actual porous media, which is quite challenging in this modelling approach because these models simplify the pore geometry. Also, the representation of microscopic features is difficult to be described by pore network models explicitly. In both naturally occurring or man-made systems, the pore structures can be significantly modified during dissolution and precipitation in porous media. Using reactive transport modelling on these occasions has been very successful. Finally, a summary table of the different modelling approaches is presented below (Table 1) with their capabilities, applications, and limitations.

**Table 1:** A summary of the different modelling methods

Modelling scale	Methods	Capabilities	Limitations
Pore scale	Level set	1.Coupling flow, transport and evolution of porous media properties 2.Applicable to large-scale natural and man-induced processes 3.Simulation of complex surface motions without domain parametrisation	1.Demands high computing time and limited pore volumes 2.The interface is constrained by the defined length function
	Lattice Boltzmann	1.Coupling flow, transport and evolution of porous media properties 2.Applicable to large-scale natural and man-induced processes 3.Able to simulate multicomponent transport problems with large chemical species 4.Simple calculation procedure 5.Efficient when implementing for parallel computation 6.Robust in handling complex geometries	1.Schemes with higher dimensions with more velocity directions are computationally expensive 2.There is no consistent thermo-hydrodynamic scheme 3.High-Mach number flows are difficult to implement
	Finite element method	1.Coupling flow, transport and evolution of porous media properties 2.Applicable to large-scale natural and man-induced processes 3.Able to model complex geometries 4.Provide high accurate results even for very complex geometries 5.Ease of incorporating boundary conditions 6.Material heterogeneity can be easily implemented 7.Ability to implement higher-order elements	1.High computing time for complex geometries with fine meshes 2.Accuracy of the results is highly dependent on mesh size
	Smoothed particle hydrodynamics (SPH)	1.Coupling flow, transport and evolution of porous media properties 2.Applicable to large-scale natural and man-induced processes 3.Triviality of treating interfacial problems 4.Advection, diffusion and reaction equations in the Lagrangian framework are reduced to diffusion and reaction equations 5.Numerical diffusion is eliminated 6.Provide superior advantage over grid-based methods when simulating multiphase or multicomponent transport problems	1.High computation cost compared to grid-based methods 2.Difficulty in using higher-order discretisation schemes unlike mesh-based methods
Mesoscale	Pore network modelling (PNM)	1.Ease of implementing material heterogeneity 2.Provides an affordable computational tool and a reduced impact on the numerical simulations as the pore volume is increased unlike other techniques 3.Able to simulate multiphase and single-phase flow in porous media	1.They are constructed with simplifying assumptions of the pore geometry 2.The governing assumption can make the predictions less accurate 3.The challenge of identifying the critical features relevant to the process of interest
Macroscale	Finite volume method	1.Applicable to simulating multiphase or multicomponent reactive transport in both saturated and unsaturated porous media 2.Captures the appearance and disappearance of phases in multiphase flow problems 3.The method enforces the conservation of the field variables after discretisation 4.It can be used on unstructured grids 5.Uses arbitrary meshes for complex geometries 6.The discontinuities of coefficients are overcome, provided the mesh is chosen such that the discontinuities occur on the boundaries of the control volume 7.Computationally inexpensive and robust for highly nonlinear systems of hyperbolic equations	1.Not applicable to highly localized processes in porous media 2.Great effort is required when the geometry is not regular
	Mixed finite element	1.All capabilities of FEM mentioned above 2.Applicable to problems where the primal-based methods are impractical	1.It requires more degrees of freedom than the displacement FEM 2.Its discrete system is indefinite since the mixed variational principal is a saddle point, as a result several matrix solution methods, direct and iterative methods, cannot be used with this technique 3.Subject to numerical instabilities not observed with standard displacement methods
	Upscaled SPH	Same as SPH above	Same as SPH above
	Hybrid methods	1.Provides significant speed-up in simulations where pore-scale simulations are localized in the computational domain 2.Provides a great benefit when the interfacial region of interest is very small compared to the entire domain	The limitations of the mixed methods apply

## Data Availability

Not applicable.

## Nomenclature

$c$: Volumetric solute concentration
$$\overline{c }$$: Mean concentration
${c}^{*}$: Concentration of the solute at equilibrium
${c}^{^{\prime}}$: Normalised solute concentration
$${C}^{l}$$: Mass fraction
$D, \mathcal{D}$: Solute molecular diffusion
$${D}^{*}$$: Dispersion tensor
${D}_{l}^{0}$: Diffusion coefficient of pure water
${D}_{l}^{min}$: Matrix effective diffusion coefficient
$$Da$$: Damkohler number
${Da}_{ls}$: Damkohler number for precipitation or dissolution process
${D}_{eff}$: Effective diffusivity tensor
${e}_{i}$: The *i*th direction dependent on the lattice type
${f}_{i}$: Probability distribution function
${f}_{i}^{eq}$: Equilibrium distribution function
$h$: Small increment in distance
${J}^{j}$: Flux of *j* component
${k}_{r}$: Reaction rate constant
$$k$$: Permeability tensor
$${k}_{0}$$: Initial permeability
$$K$$: Dimensionless quantity
${K}_{c}$: Stoichiometric coefficient of the reaction
${K}_{e}$: Effective reaction rate
${k}_{eq}$: Effective equilibrium constant
$${K}_{sp}$$: Solubility product
${L}_{c}$: Macroscopic length scale
${l}_{l}$: Pore-geometry length scale
$m$: Moles of mineral
${m}_{i}$: Mass of particle *i*
$n$: Unit normal vector
$${n}_{c}$$: Cementation factor
${n}_{i}$: Particle density of particle *i*
$N(x)$: Number of liquid SPH particles contained in the representative volume
${N}_{c}$: Number of primary species
${N}_{m,r}$: Number of reactive minerals
${N}_{m}$: Number of minerals under kinetics conditions
${N}_{x}$: Number of secondary species
$$p, {p}_{0}$$: Fluid pressure
Pe: Peclet number
$Q$: Activity products relating to the concentrations of species
$${q}_{n}$$: Flow rate
${r}_{i}$: Position vector of particle $i$
${r}_{m}$: Rate of precipitation or dissolution of mineral m
${R}_{surf}^{j}$: Surface reaction source-sink term for *j* component
$S$: Reactive surface of the mineral exposed to the fluid reactants
${S}_{0}$: Initial reactive surface area
$\Delta t$: Discrete-time step
$v$: Fluid velocity vector
$V$: Representative volume element
$${V}_{s}$$: Solution volume
$${V}_{i}$$: Interface velocity
${w}_{i}$: Weights for the distribution function along the *i*th direction
$W$: SPH smoothing kernel
$${W}_{x,{r}_{0}}\left({y}_{b}\right)$$: Weighting function
$x$: Position vector of the interface
$\Delta x$: Distance between two lattice nodes
${y}_{b}$: Position vector of particle $b$
$\varepsilon$: Length-scale parameter
$${\rho }_{F}$$: Fluid density
$${\rho }_{s}$$: Solid density
$${\rho }_{l}$$: Liquid density
$\kappa$: Local reaction rate constant
$\mu$: Fluid dynamic viscosity
$\theta , \eta$: Empirical parameters in Eq. (13)
$\parallel \parallel$: Sign showing the magnitude of the corresponding vector
${\varphi }_{i}$: Mineral volume fraction
${\varphi }_{NR}$: Volume fraction of the non-reactive minerals
$${\Omega }_{j}$$: Saturation ratio
${\Omega }^{BGK}$: Lattice Boltzmann BGK collision operator
$$\mho$$: Averaging volume
$\tau$: Lattice Boltzmann relaxation time
$${\tau }_{l}$$: Tortuosity
${\Xi }^{l}$: Dimensionless surface concentration of species $l$ formed on the surface
$$\phi (\mathrm{x},t)$$: Contour function
$$\phi$$: Total porosity
$${\phi }_{e}$$: Effective porosity
$${\phi }_{crit}$$: Critical porosity
$${\phi }_{0}$$: Initial porosity
$$\chi$$: Closure variable
$\mathcal{K}$: Reaction rate constant for a surface reaction
${\Gamma }_{sl}^{T}$: Solid–fluid interface
$${\lambda }_{j}$$: Species *j* mobility
